# Seasonal dynamics of typhoid and paratyphoid fever

**DOI:** 10.1038/s41598-018-25234-w

**Published:** 2018-05-02

**Authors:** Neil J. Saad, Victoria D. Lynch, Marina Antillón, Chongguang Yang, John A. Crump, Virginia E. Pitzer

**Affiliations:** 1 Department of Epidemiology of Microbial Diseases, Yale School of Public Health, Yale University, New Haven, Connecticut United States of America; 2 Centre for International Health, University of Otago, Dunedin, New Zealand

## Abstract

Typhoid and paratyphoid fever may follow a seasonal pattern, but this pattern is not well characterized. Moreover, the environmental drivers that influence seasonal dynamics are not fully understood, although increasing evidence suggests that rainfall and temperature may play an important role. We compiled a database of typhoid, paratyphoid, or enteric fever and their potential environmental drivers. We assessed the seasonal dynamics by region and latitude, quantifying the mean timing of peak prevalence and seasonal variability. Moreover, we investigated the potential drivers of the seasonal dynamics and compared the seasonal dynamics for typhoid and paratyphoid fever. We observed a distinct seasonal pattern for enteric and typhoid fever by latitude, with seasonal variability more pronounced further from the equator. We also found evidence of a positive association between preceding rainfall and enteric fever among settings 35°–11°N and a more consistent positive association between temperature and enteric fever incidence across most regions of the world. In conclusion, we identified varying seasonal dynamics for enteric or typhoid fever in association with environmental factors. The underlying mechanisms that drive the seasonality of enteric fever are likely dependent on the local context and should be taken into account in future control efforts.

## Introduction

Typhoid and paratyphoid fever are febrile illnesses, exclusive to humans, caused by *Salmonella enterica* serovar Typhi (*S*. Typhi) and *Salmonella enterica* serovars Paratyphi A, B and C (*S*. Paratyphi), respectively, and are together referred to as enteric fever. Globally, the pathogens are estimated to cause 6.9 to 48.4 million cases annually with most of this burden occurring in Asia and Africa^1,2^.

In several Asian and African countries, enteric fever tends to follow a seasonal pattern, with a regular recurrence of peak incidence around the same time each year. In Bangladesh, Nepal, and Cambodia (South and Southeast Asia), incidence peaks around May to October^3–5^, while across Africa it presents in a range of seasonal patterns. In Blantyre, Malawi, a distinct seasonal cycle was observed with a peak in March-June following the rainy season^6,7^, but more complex dynamics were found in Nairobi, Kenya^8,9^. In Cameroon and the Democratic Republic of Congo, a more constant occurrence of the disease has been described^10,11^.

*S*. Typhi and *S*. Paratyphi are transmitted through ingestion of fecally contaminated food or water^1,2^. Variation in factors that influence these modes of transmission, such as host behavior or environmental factors, can result in fluctuating or complex seasonal dynamics^12^. Indeed, a recent analysis of drinking water sources in Kathmandu, Nepal, found *S*. Typhi and *S*. Paratyphi A DNA present in all sources throughout the year, with peak prevalence following the monsoon season^13^.

The drivers responsible for the seasonal occurrence of enteric fever are not fully understood. Some evidence suggests that rainfall and temperature may play an important role. In Dhaka, Bangladesh, typhoid fever was positively associated with higher temperature, higher rainfall, or water level of nearby water bodies^14^. Similarly, in the Hongta District of the Yunnan Province, China, the number of cases increased with higher rainfall and clustered spatially in the vicinity of markets and rainwater canals, which were used as open sewers and prone to flooding^15^.

A better understanding of the seasonal dynamics and environmental correlates of typhoid and paratyphoid fever could help to identify the predominant drivers of transmission, thereby aiding the evaluation of disease surveillance and control efforts. Furthermore, understanding the mechanisms through which temperature and rainfall are associated with enteric fever incidence may help to pinpoint important sources of infection. We examined the seasonal pattern of enteric fever globally, determined the mean time of the seasonal peak on an annual cycle, quantified the seasonal variation and evaluated the potential environmental drivers of these seasonal dynamics.

## Methods

### Data sources and data extraction

We compiled a database of typhoid, paratyphoid, or enteric fever seasonal dynamics by identifying eligible articles and relevant data using two strategies. First, we conducted a systematic review by searching EMBASE, MEDLINE, Global Health, PubMed, and Web of Science for eligible articles (Table S1). Second, we searched for eligible articles and unpublished datasets in our personal library.

Eligible articles investigated the seasonality of typhoid, paratyphoid, or enteric fever and/or its relationship with climate. Search terms are listed in Table S1. Studies were excluded if the study duration was less than two years, if the disease data were not reported at a monthly or a finer resolution, and if the data were obtained from conference abstracts, case reports, or studies with the occurrence of less than 40 cases per year to avoid spurious results. Cases were ascertained by microbiological culture of blood, bone marrow, urine or stool, serological tests (Widal test and Typhidot), or based on clinical suspicion. Diagnosis by bone marrow and blood culture are highly specific and at least moderately sensitive, but most other means of diagnosis have major shortcomings of both sensitivity and specificity. Therefore, we assessed the robustness of our findings to the case definition in a sensitivity analysis.

We compiled the disease datasets by extracting the monthly occurrence of cases from eligible articles. We then calculated the average number of disease occurrences (separately for typhoid and paratyphoid fever, or enteric fever if not specified) for each month across the study period, and then calculated the relative monthly occurrence across the calendar year (i.e., the percentage of cases for each month of the year). We also extracted information on the characteristics of the study population, study design, and ascertainment of disease.

We complemented each disease dataset with location-specific geographic information on the study site (longitude and latitude). For country-level data, we used a single measure of central tendency. For subnational level data, we also included the altitude of the study site and the average monthly temperature and rainfall during the study period, which we obtained from a gridded climate dataset (CRU TS3.24.01)^16^.

The datasets generated and/or analysed during the current study are available from the corresponding author on reasonable request.

### Statistical analysis

We assessed the seasonal dynamics of enteric fever by examining the relative monthly occurrence of cases by geographic region (defined by the United Nations statistics division^17^) and latitude using an existing classification that categorizes latitude into five roughly equal divisions (70–36° North, 35–11°North, 10° North-10° South, 11–35° South, 36–70° South), which has been previously used to examine disease seasonality^18^. Second, we evaluated two seasonal metrics: the mean timing of the seasonal peak and the seasonal variation. We estimated the mean timing of the seasonal peak in enteric fever by determining the mean month of annual distribution, referred to as the centre of gravity, for each dataset using circular statistics; we obtained 95% confidence intervals (CIs) using bootstrap resampling^19^. We quantified the seasonal variation for each dataset by calculating the seasonal intensity (peak/mean) and amplitude ([peak-trough]/peak). We evaluated potential trends with the Pearson correlation coefficient.

To examine the potential environmental drivers of the seasonal dynamics, we examined the relationship between rainfall and temperature and disease occurrence and seasonal variation in studies with subnational data. First, we calculated the Pearson’s correlation coefficient between climatic factors, including monthly rainfall and temperature, and enteric fever cases at different lags (from 0 to 11 months) for the annual average monthly time-series to determine the lag corresponding to the strongest association. Second, we regressed the seasonal variation against the average location-specific rainfall and temperature, the variation in average rainfall and temperature, and the study location’s elevation for all datasets with subnational data.

We evaluated potential sources of variation in the main analysis by conducting subgroup analyses by disease (typhoid or enteric fever), spatial scale (national or subnational level data), and temporality (historical or contemporary data, with 1990 considered the cut-off to yield a similar number of studies during both periods). Moreover, we conducted a sensitivity analysis by repeating the main analysis only among datasets in which cases were diagnosed by blood or bone marrow culture. As a secondary analysis, we compared the seasonal pattern and seasonal metrics (mean timing of the peak, intensity and amplitude) for studies that concurrently recorded data on typhoid and paratyphoid fever across the study period.

All analyses were conducted in R 3.2.4 (R Foundation for Statistical Computing, Vienna, Austria). We used the ggmap package^20^ to obtain the latitude and longitude, the rgbif package^21^ to obtain altitude, and the circular statistics package^22^ to determine the centre of gravity and 95% CI. Statistical significance was defined as P < 0.05.

## Results

### Characteristics of included datasets

Our systematic review yielded 58 articles, containing 113 datasets, and 1 dataset from our collaborators (Figure S1). These datasets covered 33 countries across five regions; the majority, 63 (55%), were from Europe, with 25 (22%) from Asia, 21 (19%) from Africa and the Middle East, 3 (3%) from North America, and only 2 (2%) from South America (Table S2). Enteric fever data were collected in just over half of the datasets (66, 58%), while 48 (42%) reported data separately for typhoid fever, of which 12 also concurrently reported paratyphoid fever data. For our primary analysis, we grouped together unspecified enteric fever and typhoid datasets, hereafter referred to as “enteric fever”.

**Figure 1:**
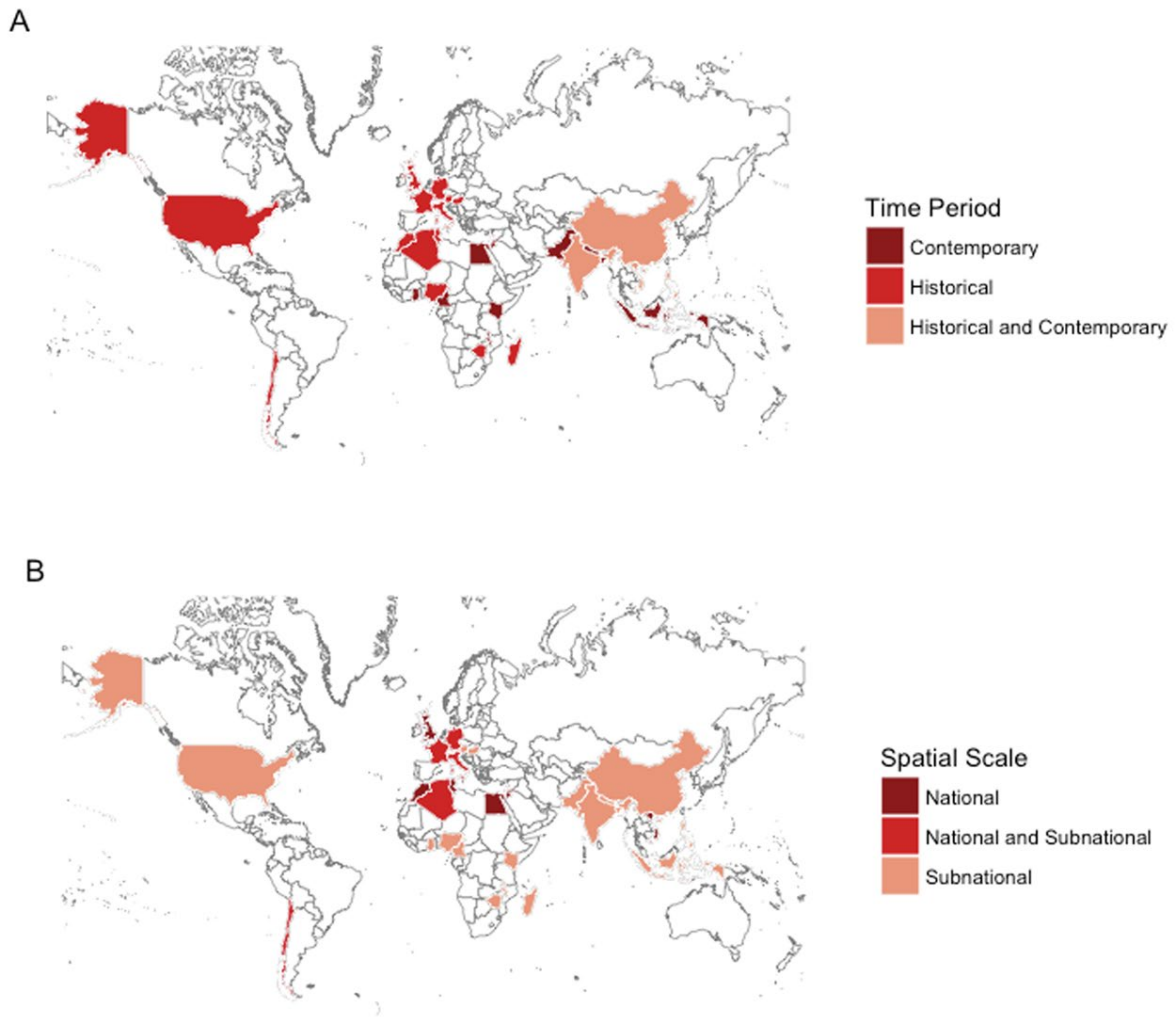
Map of countries for which data was available by (**A**) time period and (**B**) spatial resolution of the data. The Map was created in R 3.2.4 (R Foundation for Statistical Computing, Vienna, Austria) using the ggplot2^45^ and maptools^46^ packages.

The datasets differed in their epidemiological profile, with reported enteric fever incidence ranging from <1 to 980 cases per 100,000 person-years (Table S2). Figure 1A provides an overview of the time period when the data was collected, with details available in Table S2. All datasets from the Americas and Europe were collected prior to 1990, with study periods occurring between 1911 and 1986, while in Africa and the Middle East, 12 (57%) were conducted more recently (≥1990). In contrast, the vast majority of datasets in Asia (20, 80%) were recent. There was little variation in the spatial resolution of the datasets, with most (95, 83%) being collected at the subnational level (Fig. 1B).

Of 114 datasets, 84 (73.7%) contained data on cases from national or regional disease databases and 20 (17.5%) from reviewing hospital records (Table S2). The method of detection was not described in depth in 83 (72.8%) datasets, but commonly defined as according to national or regional standards. Only 16/114 datasets ascertained cases by blood culture alone, while 15/114 defined positive cases as those positive by blood culture, other cultures (bone marrow, stool or urine), and/or the Widal test; the remaining studies did not clearly specify the detection method.

### Seasonal dynamics of enteric fever

**Figure 2:**
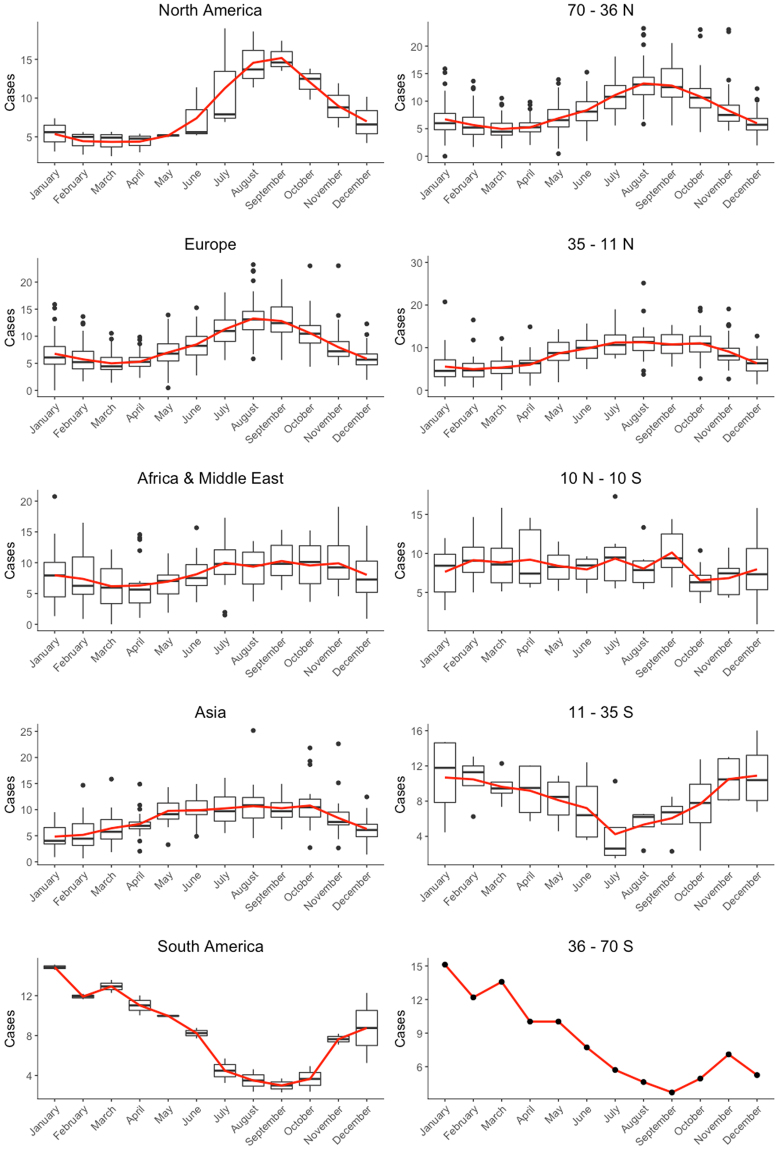
Seasonal dynamics of enteric and typhoid fever by continent and latitude. The boxplots show the percentage of cases of the different studies for each month of the year and the red line depicts the mean percentage of cases for each month of the year.

The seasonal pattern of enteric fever (Fig. 2) showed a peak in North America and Europe occurring around August-September. In Asia and Africa and the Middle East, the peak seasons spanned multiple months: from July to November in Africa and the Middle East, and from May to October in Asia. In South America, the peak period was from January to May, reflecting the different timing of the seasons in the Southern Hemisphere.

When grouped by latitude (Fig. 2), enteric fever was more likely to occur seasonally further from the equator, with a pronounced peak in August-September between 70-36°N (66 datasets, mostly European countries) and a peak period from May to October between 35-11°N (33 datasets, mostly Asian countries). Similar inverse dynamics were observed in the Southern Hemisphere (11–35°S and 36–70°S: 4 and 1 datasets, respectively), while the equatorial region (10°N-10°S: 10 datasets, of which 8 are from Africa) mostly appeared to have a constant proportion of cases throughout the year (Fig. 2), although some equatorial datasets showed a more varied pattern of cases through the year.

The seasonal pattern was not markedly influenced by spatial scale, temporality (historical or contemporary data), or disease type (typhoid or enteric fever) (Figures S2–S5), although it was not always possible to assess the influence of these factors, as some subgroups only contained one dataset. Nonetheless, there was some heterogeneity in Africa and the Middle East, primarily due to differences between datasets from North Africa and the Middle East and those in the equatorial region. Most datasets in North Africa and the Middle East studied enteric fever at the national level before 1990, whereas datasets around the equator were more recent and used subnational data.

### Seasonal metrics of enteric fever

**Figure 3:**
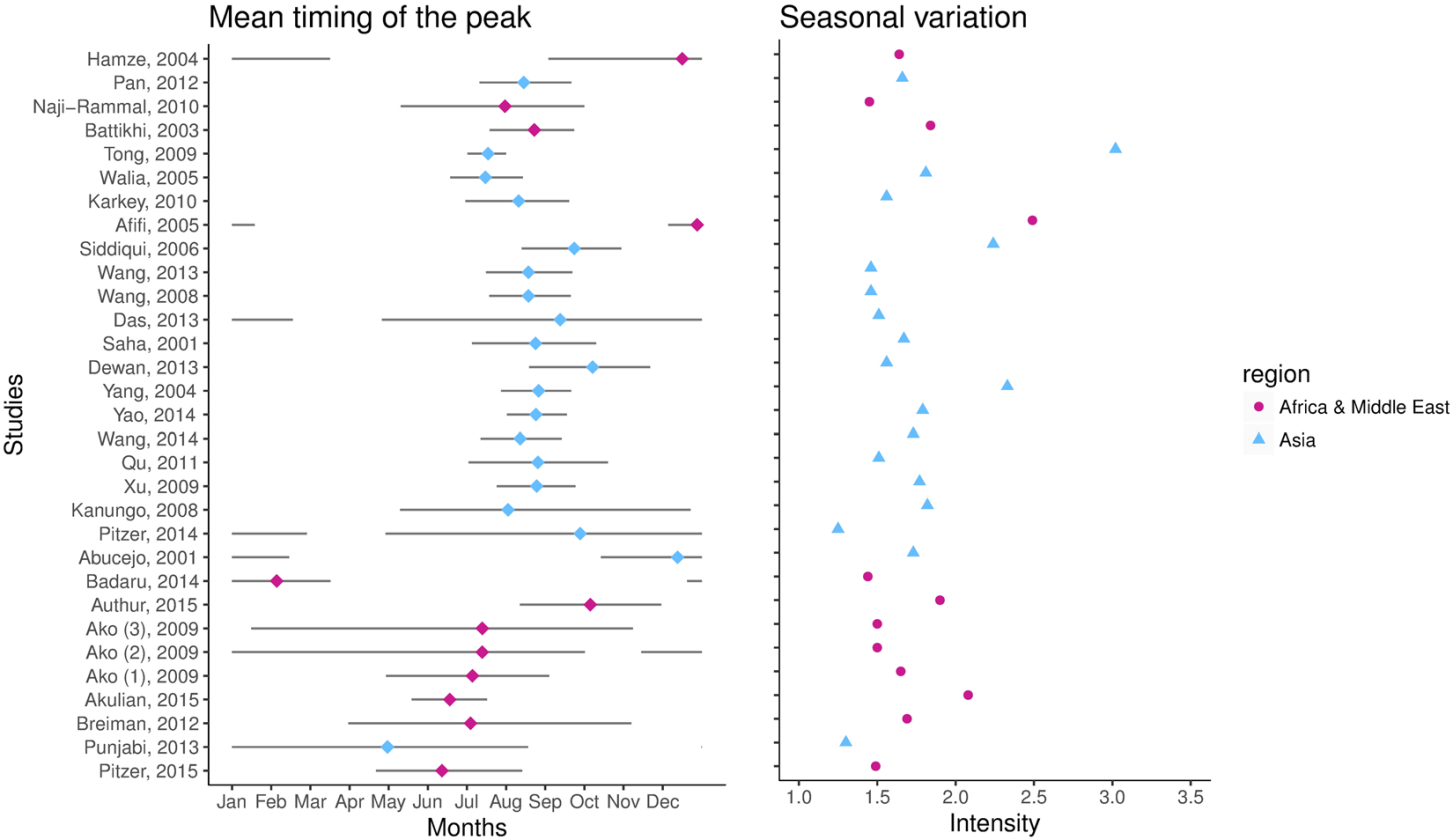
Mean timing of the peak and seasonal variation for contemporary (>1990) data on enteric and typhoid fever. Mean timing (as measured by center of gravity) is represented by the colored dots, while the lines represent the corresponding 95% confidence intervals. The seasonal variation is quantified by the seasonal intensity (peak/mean). Studies are ordered by latitude, from North (top) to South (bottom) and colored by region: Africa & Middle East (Purple) and Asia (Blue).

There was a moderate but statistically significant trend (P < 0.01) in the mean timing of the peak of enteric fever, which shifted from December to May when ordered by latitude from north to south among recent studies (≥1990) (Figs 3 and S6). This trend was independent of the disease (typhoid or enteric fever) or spatial resolution of the data (Figure S5), and persisted but was less pronounced among older datasets (<1990), as there was less geographic variation (Figure S7).

On the other hand, there was no clear pattern in the seasonal intensity of enteric fever by geographic region, latitude, disease, or spatial level of the data (P > 0.05) (Figs 3 and S5, S7). This did not change when evaluating another measure of seasonal variation, the amplitude (Figure S8).

### Environmental drivers of seasonality

**Figure 4:**
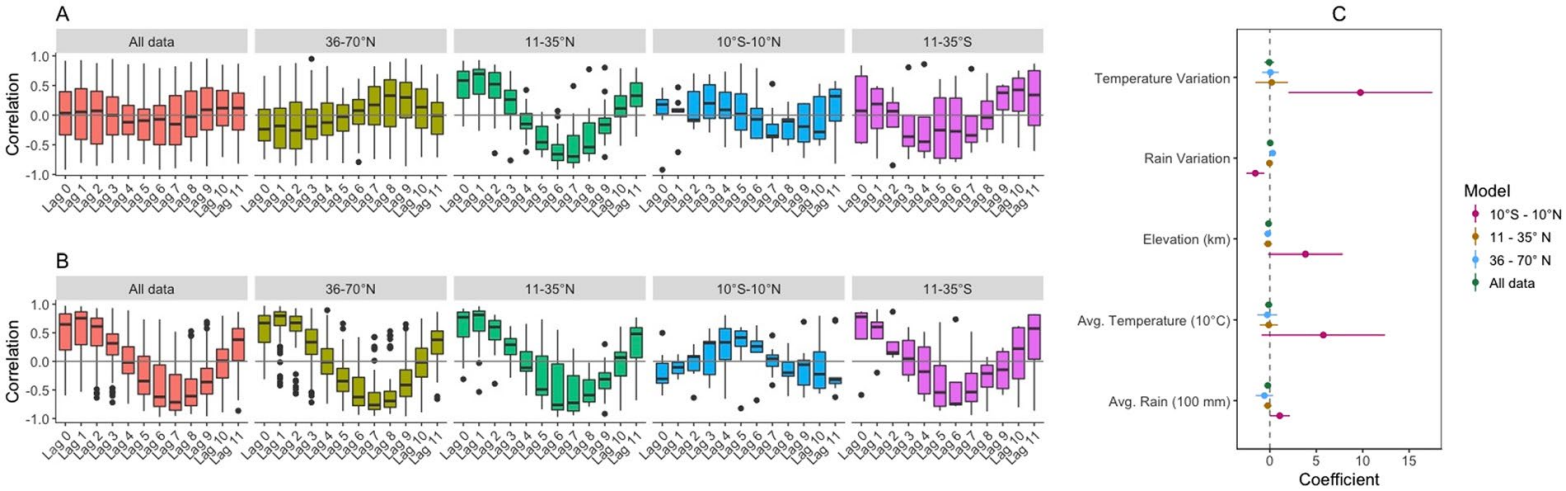
Association of environmental drivers with enteric or typhoid fever and seasonal intensity. The Pearson’s correlation coefficient between enteric or typhoid fever and (**A**) average monthly rainfall and (**B**) average monthly temperature (current and lagged (in months), up to 11 months) is summarized for all studies within a specified region. The values of the studies are represented in a boxplot, in which the whiskers represent the interquartile range (25^th^–75^th^ percentile) times 1.5. (**C**) Coefficients from the meta-regression of the seasonal variation (peak/mean) against the average location-specific rainfall and temperature, the variation in average rainfall and temperature (peak/mean), and the study location’s elevation for all datasets with subnational data are plotted. The point estimates of the regression coefficients are represented by dots, while the 95% confidence interval is indicated by a horizontal line.

Globally, there was no strong relationship between enteric fever and rainfall (Fig. 4A). However, when stratified by latitude, we found a positive correlation between enteric fever at lags of 0–2 months among settings located 35°–11°N. We found evidence of a positive correlation between temperature and enteric fever at lags of 0–2 months, except in the equatorial region (Fig. 4B).

Temperature and rainfall explained less than a tenth of the variation in seasonal intensity at the ecological level (R^2^ = 0.07), and none of the environmental factors were strongly associated with the seasonal intensity or amplitude globally in the regression analysis (Figs 4C, S9). However, in the equatorial region, reduced rainfall variation, increased average rainfall, and variation in temperature were associated with greater variation in the in the number of cases (Fig. 4C). We could not assess the environmental drivers in the Southern Hemisphere, as there were only four datasets.

### Comparing the seasonal dynamics and metrics of typhoid and paratyphoid fever

Twelve datasets provided concurrent data on typhoid and paratyphoid fever. Six studies were conducted in Europe or the Middle East between 1920 and 1970, while the other six were more recent datasets from Asia. Details on the epidemiological profile of typhoid and paratyphoid fever are provided in Table S3.

**Figure 5:**
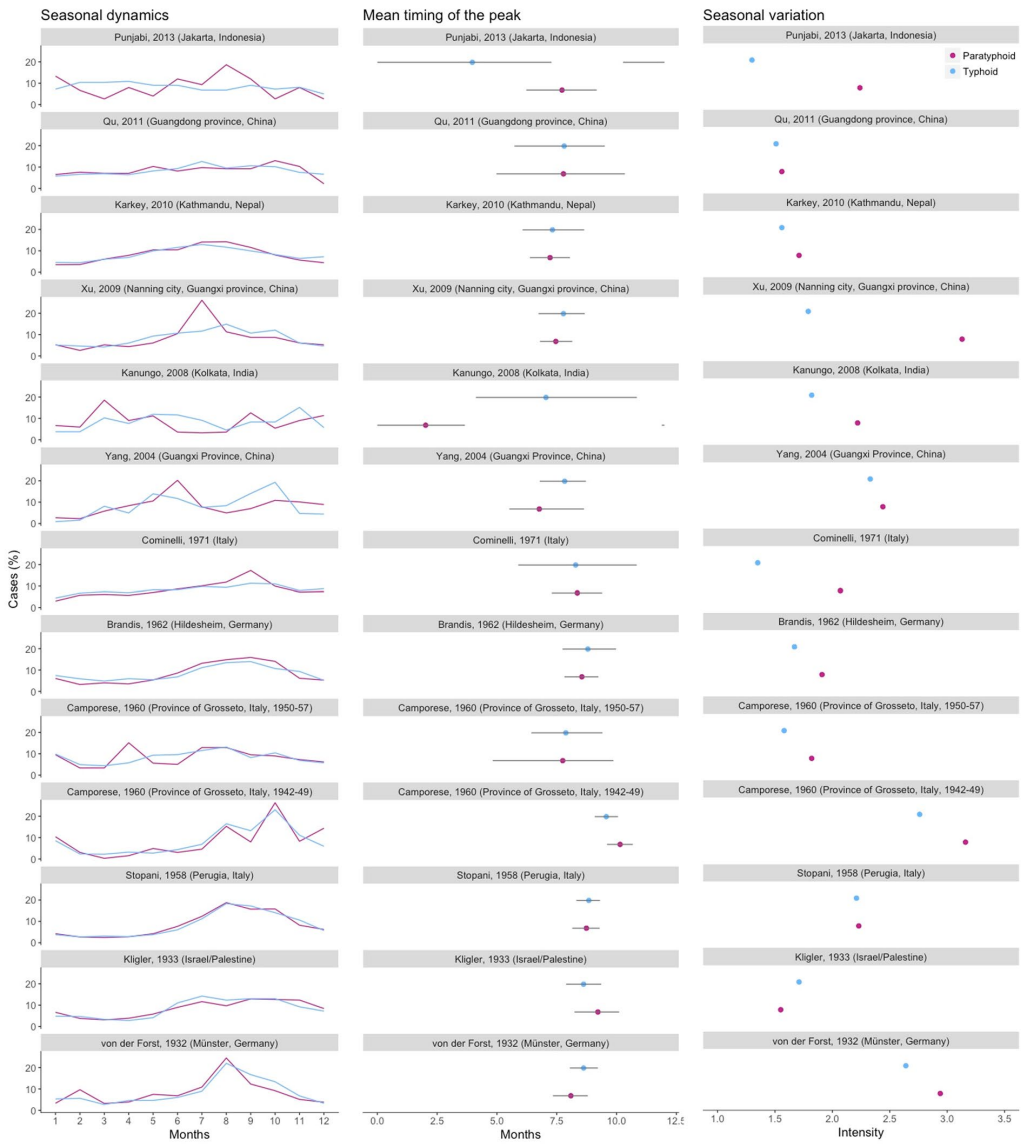
Seasonal dynamics, mean timing of the peak and seasonal variation for studies with typhoid and paratyphoid data. On the left, the percent of annual cases occurring in each month are plotted for paratyphoid fever (purple) and typhoid fever (blue). In the middle, mean timing (as defined by center of gravity) is represented by the dots, while the line corresponds to the 95% confidence interval. On the right, the seasonal variation (peak/mean) is plotted.

The seasonal dynamics of typhoid and paratyphoid fever appear to have become less congruent with time in some studies (Fig. 5). In older datasets, the seasonal pattern is nearly identical - the mean timing of the peak occurred simultaneously and the seasonal intensity was comparable. However, among contemporary studies, the seasonal pattern differed markedly, except in Kathmandu, Nepal, and Guangdong Province, China. The seasonal intensity was much greater among paratyphoid fever datasets in recent studies. Nonetheless, the mean timing of both typhoid and paratyphoid fever mostly still occurred contemporaneously.

### Sensitivity analysis

Only 16 datasets relied exclusively on blood culture to diagnose enteric fever, which complicated the comparison of results from the sensitivity analysis with those from the main analysis. Nonetheless, the seasonal pattern in Europe, Asia, and the Northern Hemisphere in the sensitivity analysis was consistent with that in the main analysis (Figure S10). In contrast, the patterns in Africa and the Middle East and the equatorial region were less similar and more variable. There were no datasets in North or South America that employed only blood culture for diagnosis. The clear trend in the mean timing of the peak persisted among datasets with a blood culture diagnosis (Figure S11) and shifted from October to May when ordered from north to south by latitude. Similar to the findings in the main analysis, there was no discernible pattern in the seasonal intensity of enteric fever (Figure S12). Finally, the findings regarding the environmental drivers were largely similar between the overall dataset and those with blood culture diagnosis alone (Figures S12, S13).

## Discussion

We observed a distinct seasonal pattern for enteric fever by latitude. The peak period occurred during August and September in the northern-most regions (36–70°N), with the peak period becoming wider from May through October in the 11–35°N region, until there was almost no seasonal pattern in the equatorial region. We also found evidence suggesting that a preceding peak of rainfall might be associated with the enteric fever among settings 35°–11°N and that the variation in disease in the equatorial region might be driven, in part, by variation in temperature.

As typhoid and paratyphoid fever are transmitted through ingestion of contaminated food or water, the likely drivers of the seasonal pattern are factors that influence transmission and differ by latitude. All contemporary datasets were from Africa or Asia, where the disease burden is associated with limited access to clean water and adequate sanitation^23^. These infrastructural deficiencies could be susceptible to climatic events, such as monsoons and droughts. However, the lack of a strong and consistent association with rainfall and/or temperature indicates that the important environmental drivers and routes of transmission may vary by setting and be influenced by other factors. In the following paragraphs, we discuss potential mechanisms by which climatic factors could influence the seasonal dynamics of enteric fever where such associations were apparent.

Among settings located 35°–11° N, the peak number of enteric fever cases occurs between May and October, which coincides with the monsoon season in many Asian countries. The monsoon season is marked by bursts of excessive rainfall, which are known to cause flooding^24^. Flooding has been identified as a risk factor for enteric fever and is thought to cause the mixing of drinking water sources with open sewers that contain fecal matter^14,25–27^. This is consistent with rainfall predating the peak in enteric fever in the observed data. In contrast, countries in the Middle East, some also located between 35°–11° N, experience a dearth of rainfall during the peak timing of enteric fever^28^. Therefore, what might explain the peak timing in these countries is the consumption of contaminated water, drinks or food in search of refreshment during the warmest months of the year^29^. It is also possible that greater consumption of rainwater, which is less contaminated with human feces than surface water, during the rainy season might mitigate disease occurrence in these Middle Eastern settings.

The lack of a clear association between rainfall and enteric fever incidence in other regions might be explained by the changing availability of microbiologically safe water. While increased rainfall could lead to greater contamination of rivers that are often used for bathing, defecating, and refuse as well as the primary source of water for drinking and cooking^30,31^, higher rainfall could also reduce the occurrence of disease due to the consumption of less contaminated rainwater. Moreover, limited supplies of clean water during hotter, drier periods could lead to water scarcity and force individuals to consume contaminated water^32^.

The association between enteric fever and temperature was more consistent across regions. There was a positive correlation between temperature and enteric fever at lags of 0–2 months, except in the equatorial region. This could be explained by the improved growth of *S*. Typhi and *S*. Paratyphi in warmer conditions. Increased temperature results in exponential growth of the bacteria, particularly on food, which has been found to plateau around 20–25 °C^33,34^.

In the equatorial region (10°N-10°S), we found that increased variation in temperature and less variation/higher average rainfall is associated with greater variation in enteric fever. While climatic conditions are generally more constant year-round in this region, this suggests that in those settings where there is more variation in temperature over the course of the year (and higher average rainfall), there is also more variation in enteric fever incidence. Hence, similar associations as those seen in the correlation analysis likely exist in some, but not all, equatorial settings.

Evaluating the potential mechanisms of the seasonal dynamics in the Southern Hemisphere was complicated by the availability of a single contemporary dataset. However, studies from the late 1970’s in Chile unearthed another important potential mechanism of contamination—agricultural practice, which closely coupled with climatic seasons. In Chile, a marked increase in cases from November to April coincided with the irrigation season; it was subsequently discovered that farmers irrigated their crops with wastewater, which was likely contaminated^35,36^. This practice might provide an alternative explanation responsible for some of the seasonal variation in typhoid in other regions as well, as shown by recent studies in Morocco^37,38^.

We found that the dynamics of typhoid and paratyphoid fever have become less congruent with time, except in Kathmandu, Nepal and Guangdong Province, China. In Kathmandu, the clinical presentation of typhoid and paratyphoid fever are indistinguishable^39^, which might provide an explanation for the similar dynamics. However, the dynamics of paratyphoid fever are not well understood and should be explored further^40^.

Our findings were generally robust when restricted to those with a blood culture diagnosis. However, there was a paucity of studies that relied solely on blood culture confirmation, and more data are needed to confirm our findings. In this regard, the ongoing work by consortia such as the Strategic Typhoid alliance across Africa and Asia, Surveillance for Enteric Fever in Asia Project, and Typhoid Fever Surveillance in Africa Program^41–43^ are important and will provide novel insights into the disease’s transmission dynamics.

The seasonal dynamics do not appear to vary markedly by spatial scale, except possibly in Africa and the Middle East. This might be partially explained by the fact that most datasets in North Africa and the Middle East used national level data from before 1990, whereas datasets in sub-Saharan Africa used more recent subnational data. In Asia, only two datasets used national level data, which limited our ability to draw strong conclusions on the national-level dynamics in this region. This highlights the need for data with sufficient spatial resolution to disentangle potential variation between the national and regional level.

Moreover, the underlying mechanism driving the seasonal dynamics within regions might differ. Therefore, we should be mindful of the different local climate contexts that exist within countries. For example, these underlying seasonal drivers could differ in urban and rural areas despite a high prevalence of disease in both environments^43^. While cities tend to have better infrastructure facilities, such as centrally-treated piped water and improved sanitation, those residing in many cities do not have equal access to these basic amenities, as evidenced by the numerous informal settlements that exist in cities worldwide^44^. This also underlines the importance of socioeconomic factors as the frailty of infrastructure facilities and common lack of basic amenities among informal settlements affects those most impoverished, which, in turn, places them at greater risk of disease.

Finally, we have compared the aggregate annual dynamics between studies, countries and regions, which did not enable us to assess the seasonal dynamics across multiple years. Unfortunately, few long-term time-series of culture-confirmed enteric fever with sufficient resolution exist that would enable us to investigate and compare these dynamics further. Future studies should focus on examining associations between climatic variables and culture-confirmed enteric fever incidence in settings with longstanding surveillance platforms.

## Conclusion and recommendations for future studies

In conclusion, we found distinct seasonal patterns for enteric fever by latitude. These dynamics might be explained by factors related to sources and modes of transmission, including inadequate access to clean water and sanitation, although associations with rainfall and temperature varied by region. Further work is needed to explore how socioeconomic factors may help to explain some of the different patterns we observed. Although the dynamics might be similar across geographic locations, the underlying mechanisms could be very different. Again, more multi-year datasets of culture-confirmed enteric fever cases at different spatial scales are needed. This could lead to a better understanding of the local mechanisms that drive the seasonality and transmission of enteric fever, which could aid surveillance and control efforts.

## Electronic supplementary material

Supplementary information
