# Supplementary material for: Seasonal dynamics of typhoid and paratyphoid fever
Source: Sci Rep. 2018 May 2;8:6870. doi: 10.1038/s41598-018-25234-w (PMC5932015; doi:10.1038/s41598-018-25234-w)
Supplement: Supplementary file 1 — Supplementary information [file 41598_2018_25234_MOESM1_ESM.pdf]

Seasonal dynamics of typhoid and paratyphoid fever

Supplementary Figures and Tables

Neil J. Saad[1]*, Victoria D. Lynch[1], Marina Antillon[1], Chongguang Yang[1], John A. Crump[2], Virginia E. Pitzer[1]*

[1]Department of Epidemiology of Microbial Diseases, Yale School of Public Health, Yale University, New Haven, Connecticut, United States of America

[2]Centre for International Health, University of Otago, Dunedin, New Zealand.

*****Corresponding authors

*Corresponding author contact information:

Neil J. Saad or Virginia E. Pitzer

Department of Epidemiology of Microbial Diseases

Yale School of Public Health, Yale University

60 College St, New Haven, CT, USA

Email: neil.saad@yale.edu or virginia.pitzer@yale.edu

Supplementary Figure 1. Flow chart of studies.

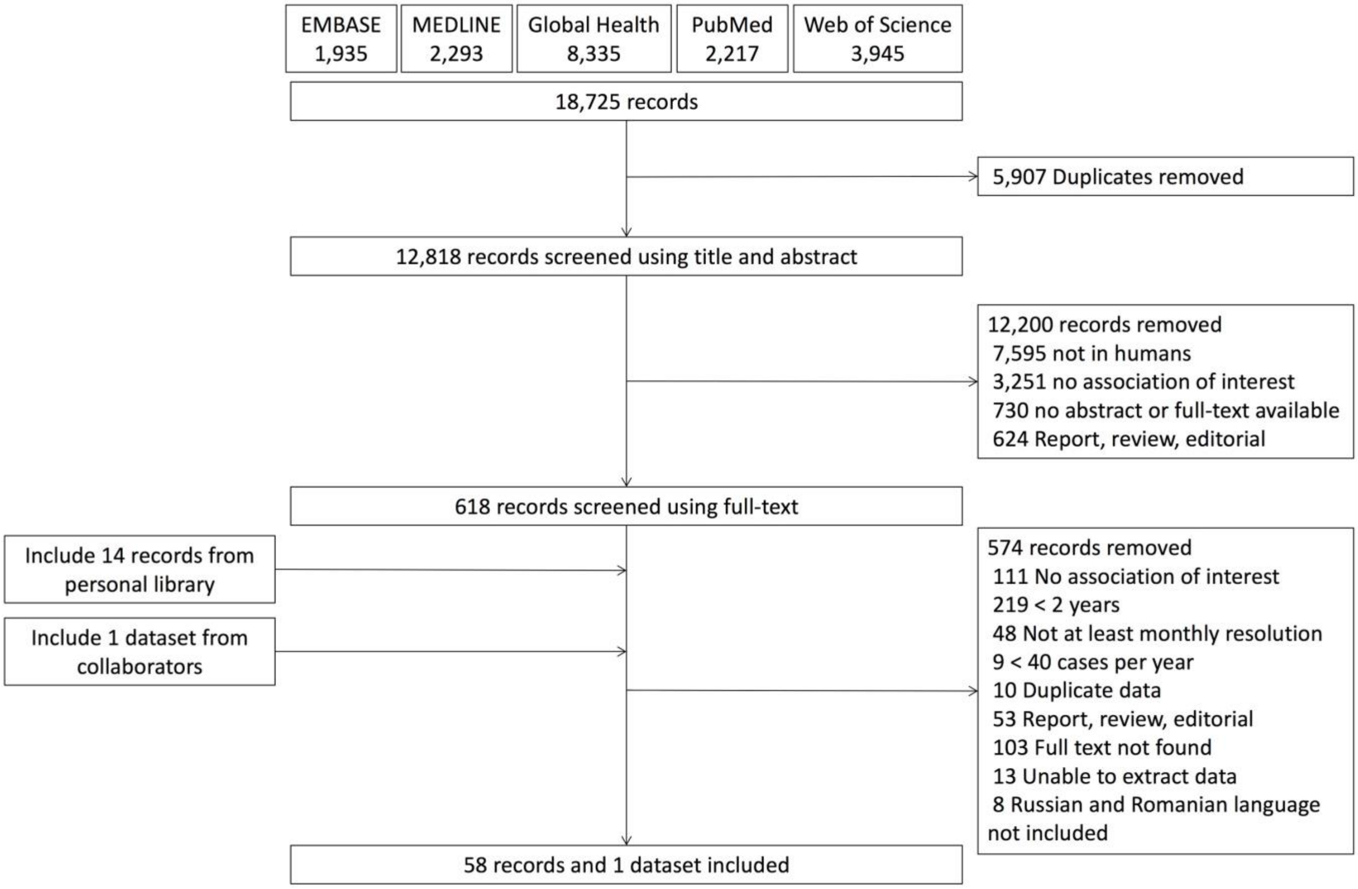

Supplementary Figure 2. Seasonal dynamics of enteric and typhoid fever by continent and latitude by spatial scale. The boxplots show the percentage of cases of the different studies for each month of the year.

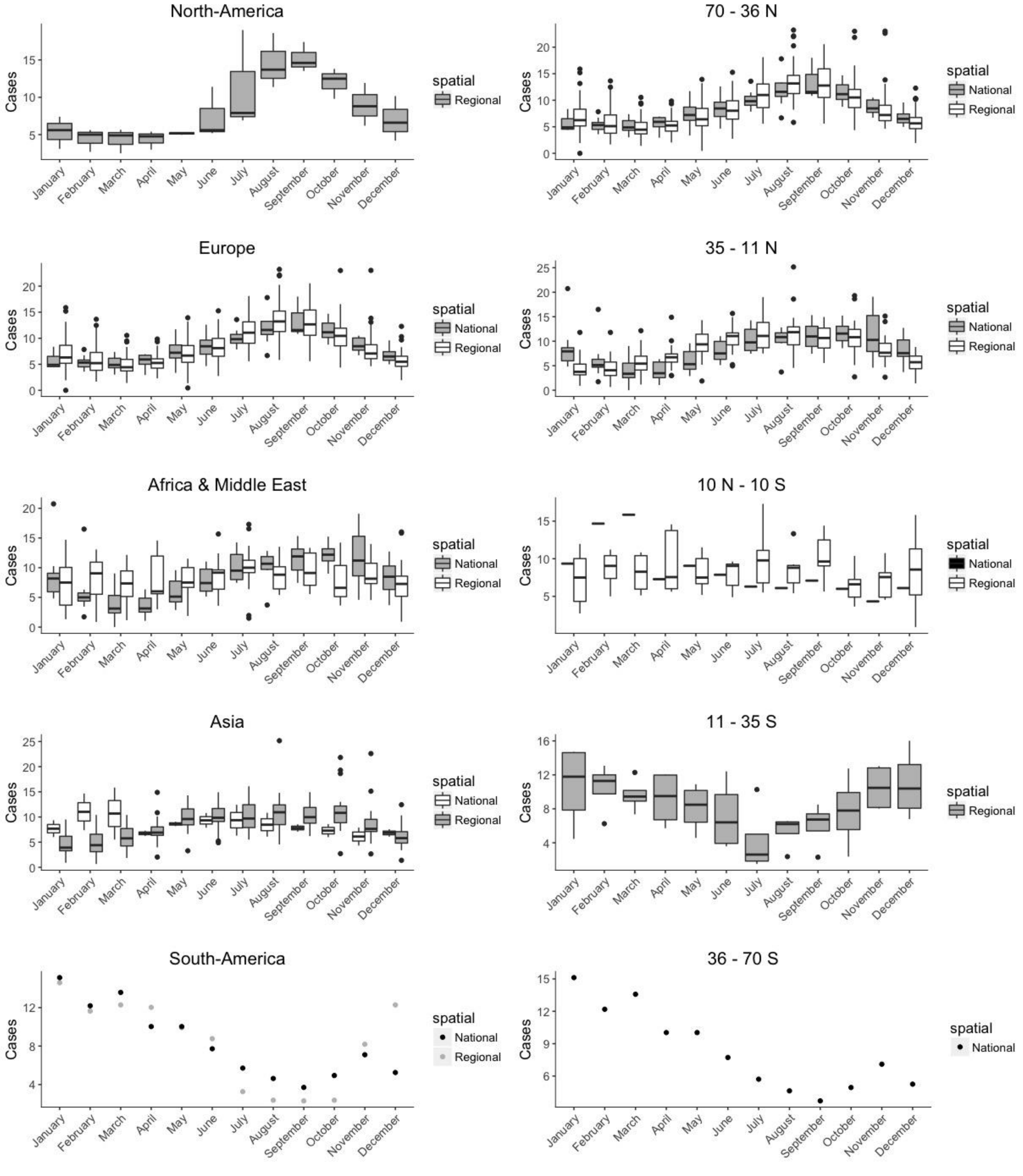

Supplementary Figure 3. Seasonal dynamics of enteric and typhoid fever by continent and latitude by temporality. Historical (<1990) and Contemporary ((≥1990). The boxplots show the percentage of cases of the different studies for each month of the year.

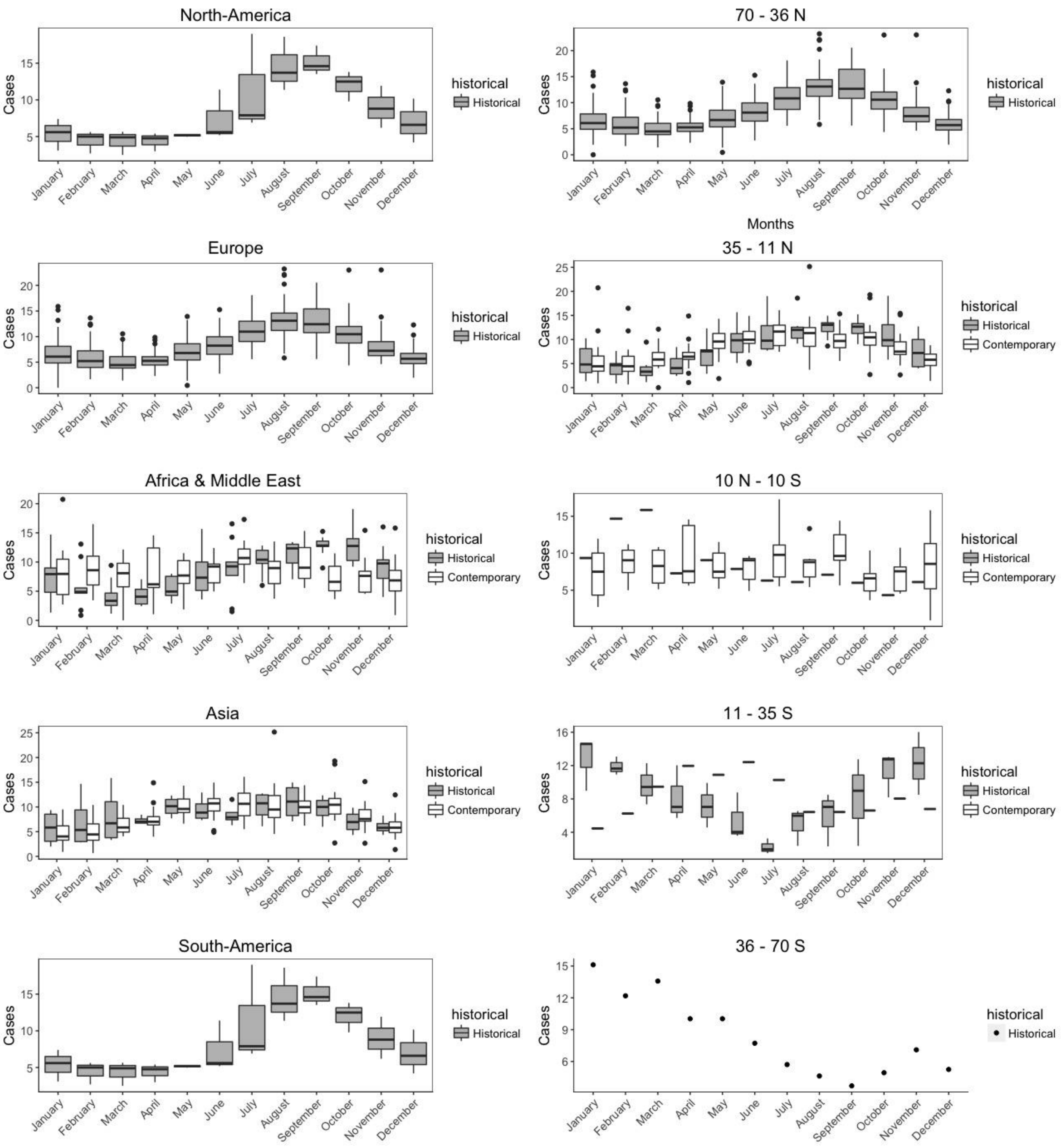

Supplementary Figure 4. Seasonal dynamics of enteric and typhoid fever by continent and latitude by disease. The boxplots show the percentage of cases of the different studies for each month of the year.

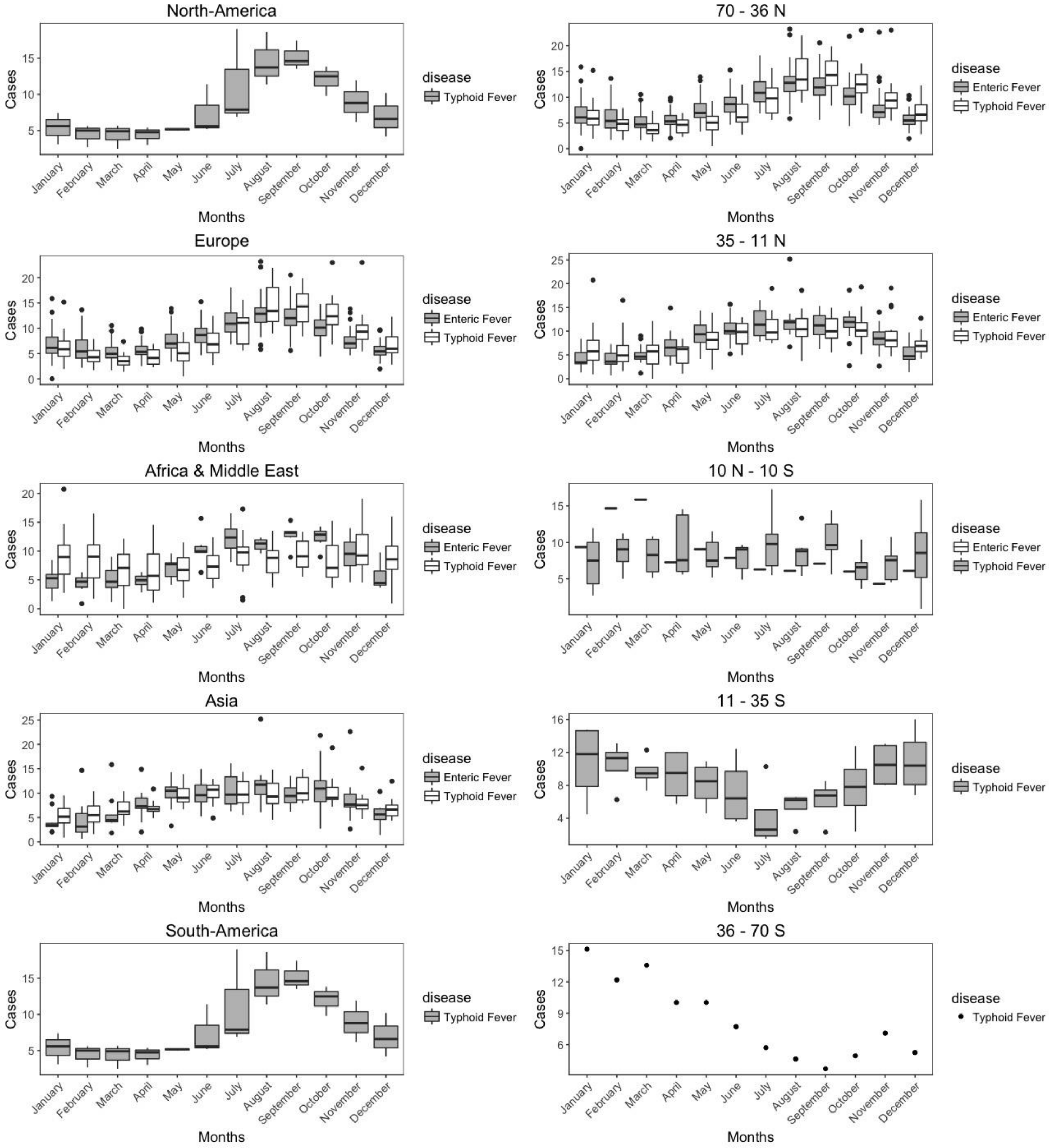

Supplementary Figure 5. Mean timing of the peak and seasonal variation for contemporary ((≥1990) data on enteric and typhoid fever by spatial scale (*top*) and disease (*bottom*). Mean timing (as measured by center of gravity) is represented by the colored dots (Africa & Middle East) or triangles (Asia), while the lines represent the corresponding 95% confidence intervals. The seasonal variation is quantified by the seasonal intensity (peak/mean). Studies are ordered by latitude, from North (top) to South (bottom) and colored by spatial scale (National (Purple) and Regional (Blue)) or disease (Enteric (Purple) and Typhoid (Blue) fever).

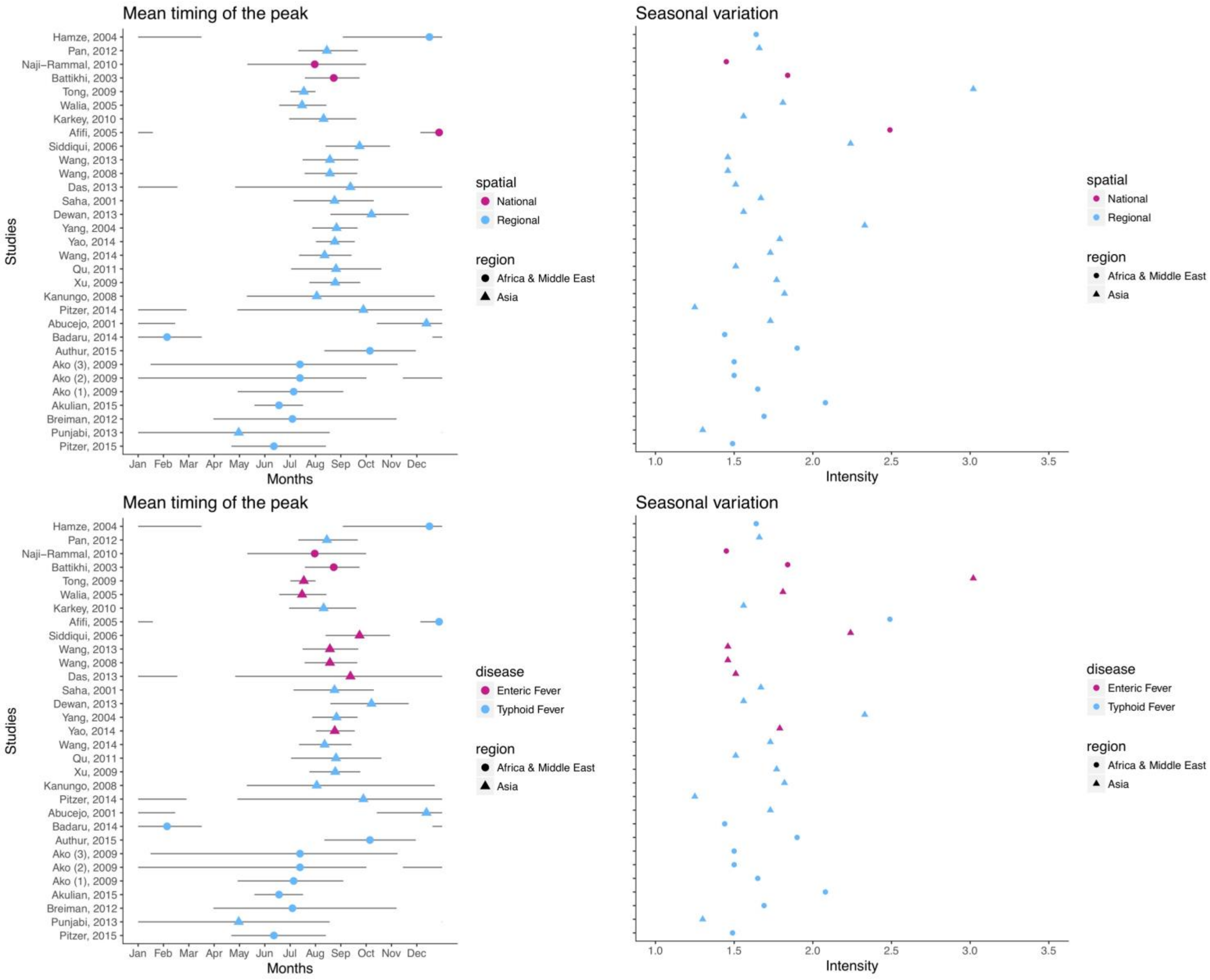

Supplementary Figure 6. Mean timing of the peak for contemporary (≥1990) data on enteric and typhoid fever plotted against latitude.

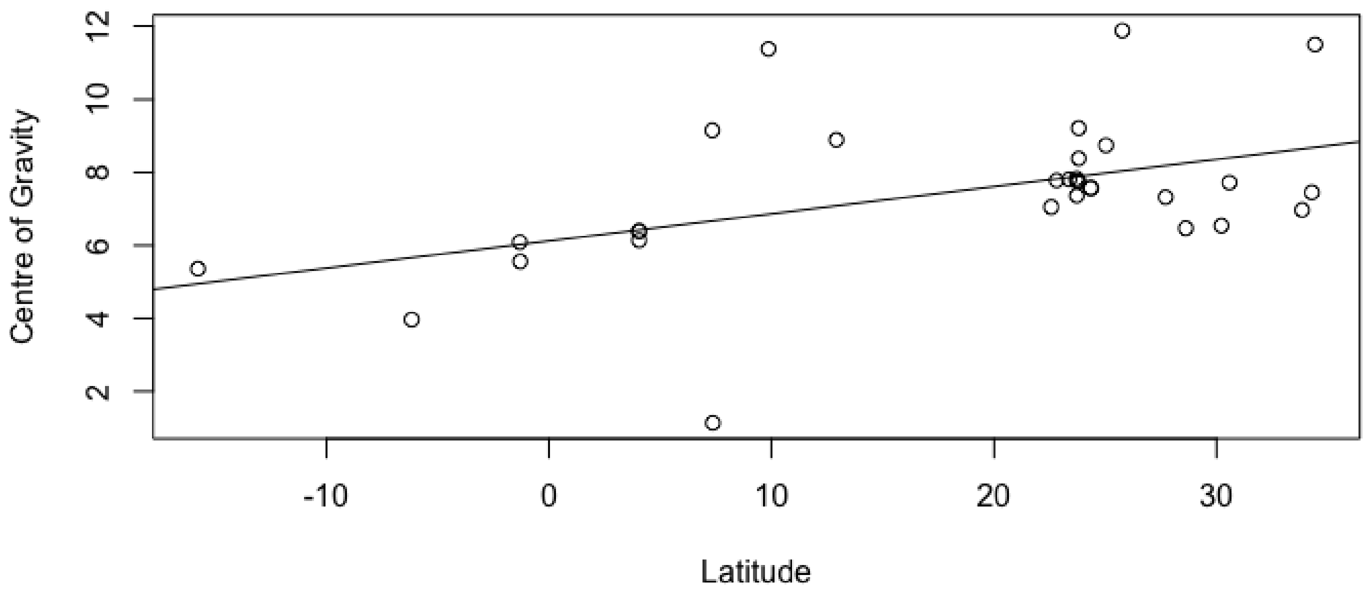

Supplementary Figure 7. Mean timing of the peak and seasonal variation for historical (<1990) data on enteric and typhoid fever. Mean timing (as measured by center of gravity) is represented by the colored dots, while the lines represent the corresponding 95% confidence intervals. The seasonal variation is quantified by the seasonal intensity (peak/mean). Studies are ordered by latitude, from North (top) to South (bottom) and colored by region: Africa & Middle East (Brown dots), Asia (Black triangle), North-America (Purple square), South-America (Blue cross) and Europe (Golden checked box).

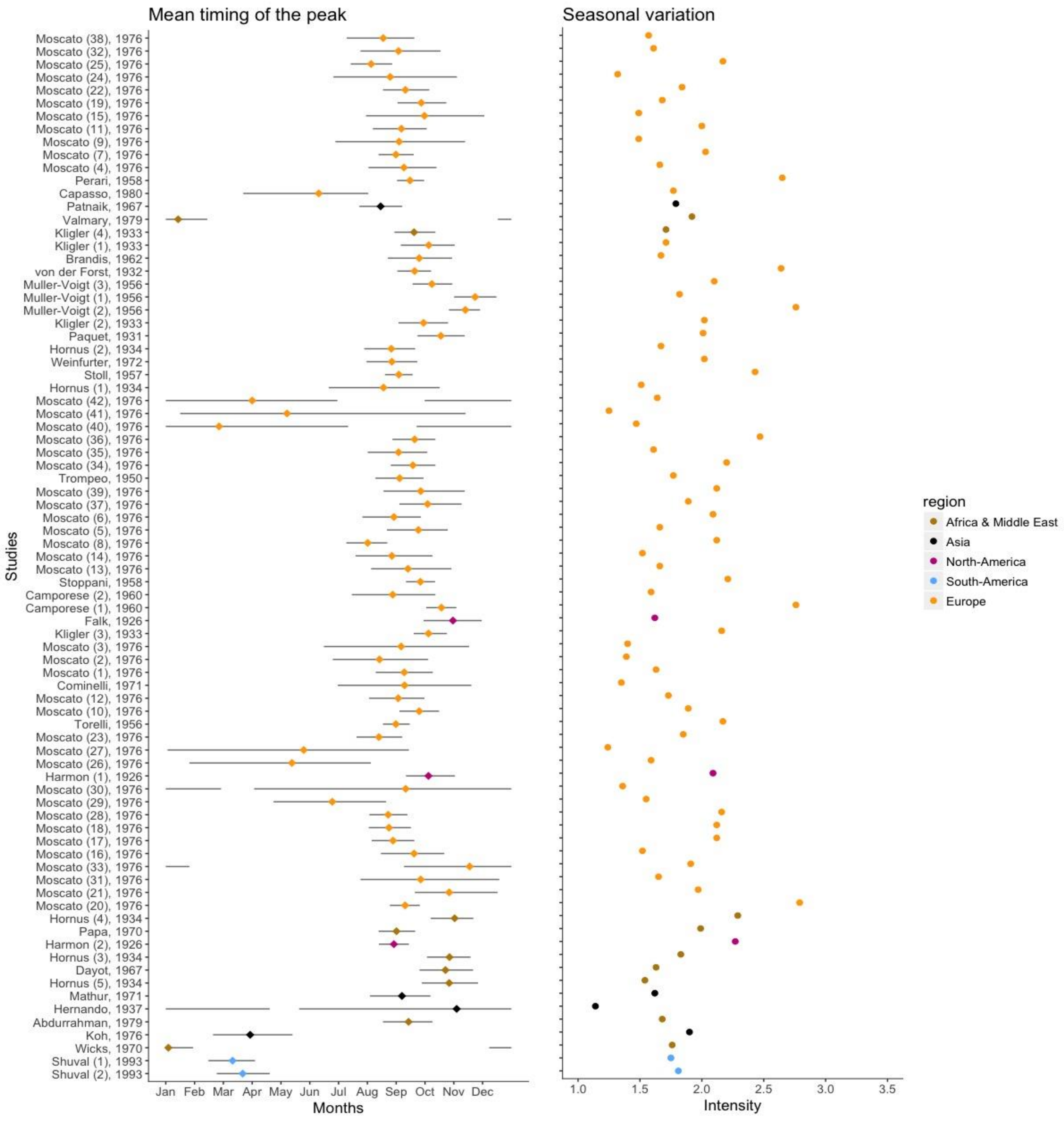

Supplementary Figure 8. Seasonal variation as measured by seasonal intensity (peak/mean) and amplitude ([peak-trough]/peak) for contemporary (≥1990) data on enteric and typhoid fever. Studies are ordered by latitude, from North (top) to South (bottom).

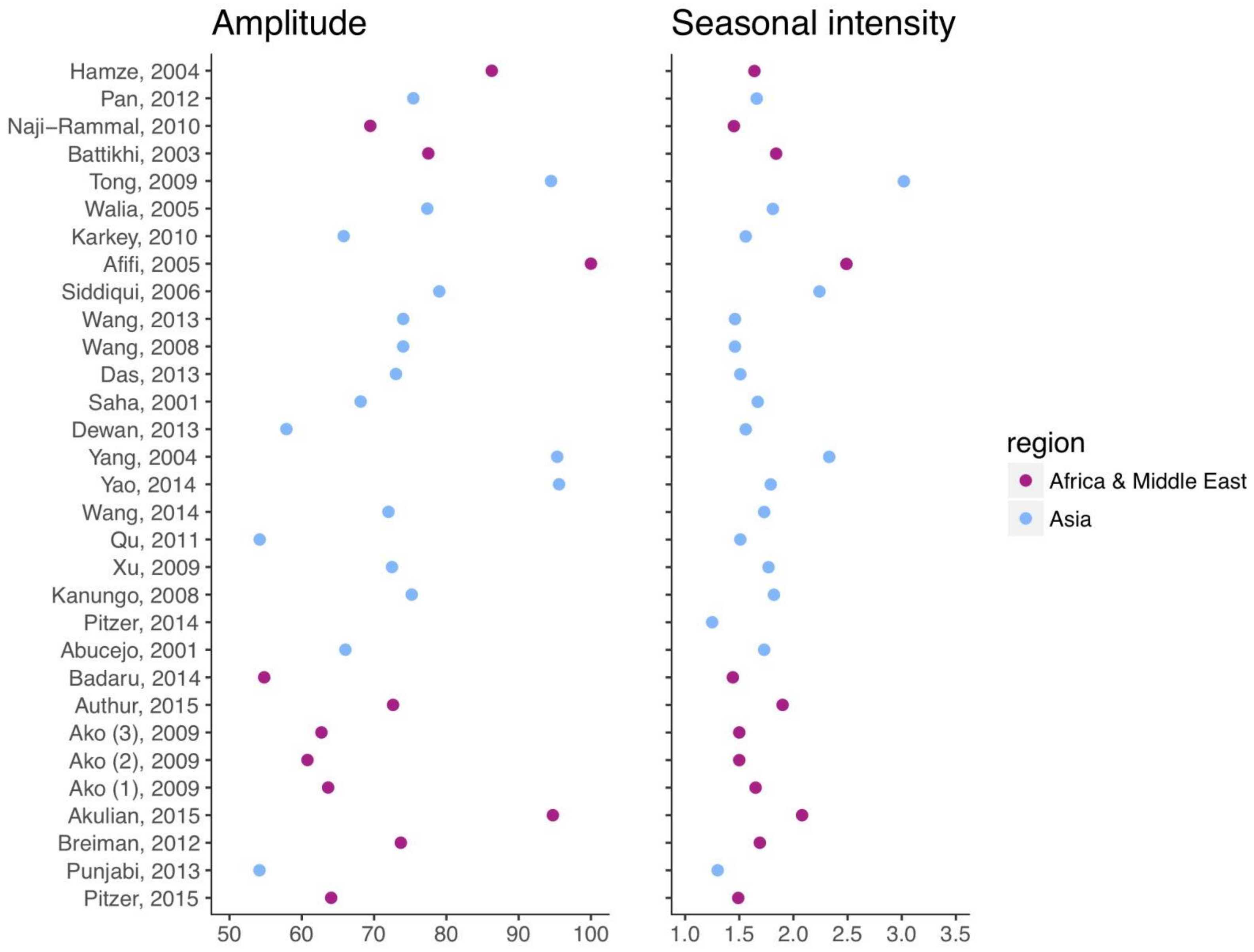

Supplementary Figure 9. Association of environmental drivers with seasonal variation as measured by seasonal intensity (peak/mean) and amplitude ((peak-trough)/peak). Coefficients from the meta-regression of the seasonal intensity (peak/mean) and amplitude ((peak-trough)/peak) against the average location-specific rainfall and temperature, the variation in average rainfall and temperature (peak/mean), and the study location's elevation for all datasets with subnational data are plotted. The point estimates of the regression coefficients are represented by dots, while the 95% confidence interval is indicated by a horizontal line.

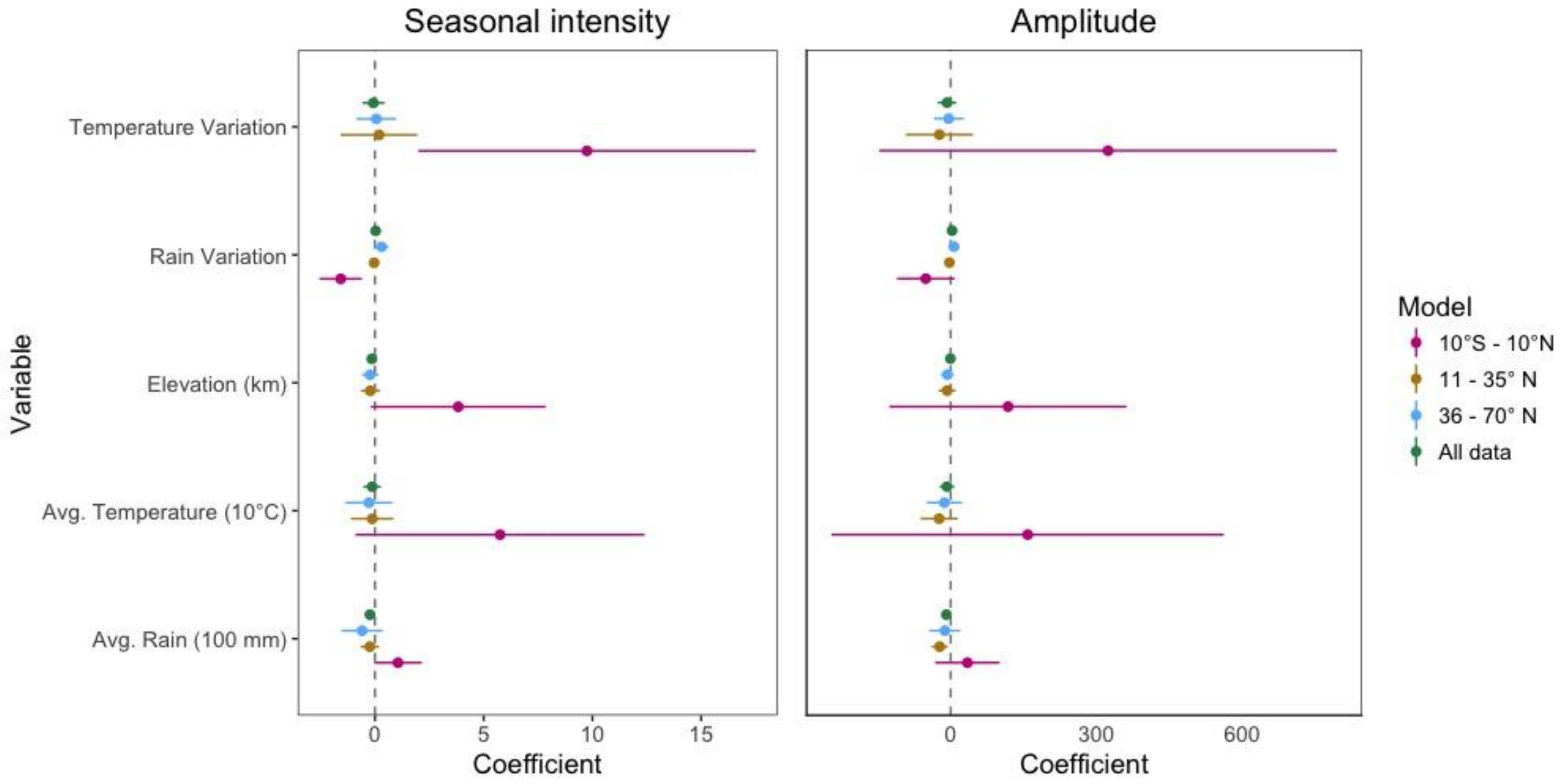

Supplementary Figure 10. Seasonal dynamics of enteric and typhoid fever by continent and latitude for data confirmed by blood culture only. The boxplots show the percentage of cases of the different studies for each month of the year and the red line depicts the mean percentage of cases for each month of the year.

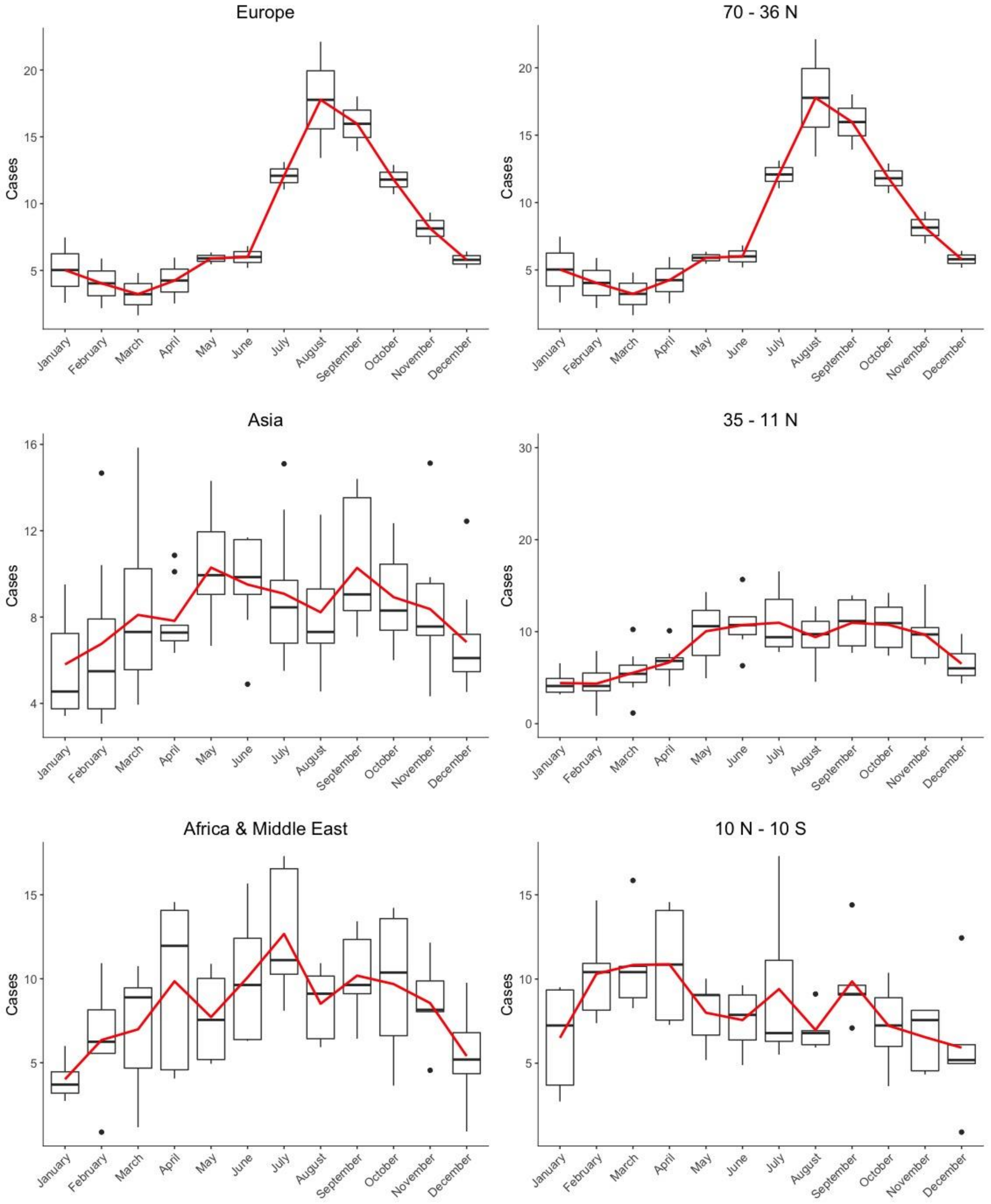

Supplementary Figure 11. Mean timing of the peak and seasonal variation for data on enteric and typhoid fever, confirmed by blood culture only. Mean timing (as measured by center of gravity) is represented by the colored dots, while the lines represent the corresponding 95% confidence intervals. The seasonal variation is quantified by the seasonal intensity (peak/mean). Studies are ordered by latitude, from North (top) to South (bottom) and colored by region: Africa & Middle East (Golden), Asia (Purple) and Europe (Blue).

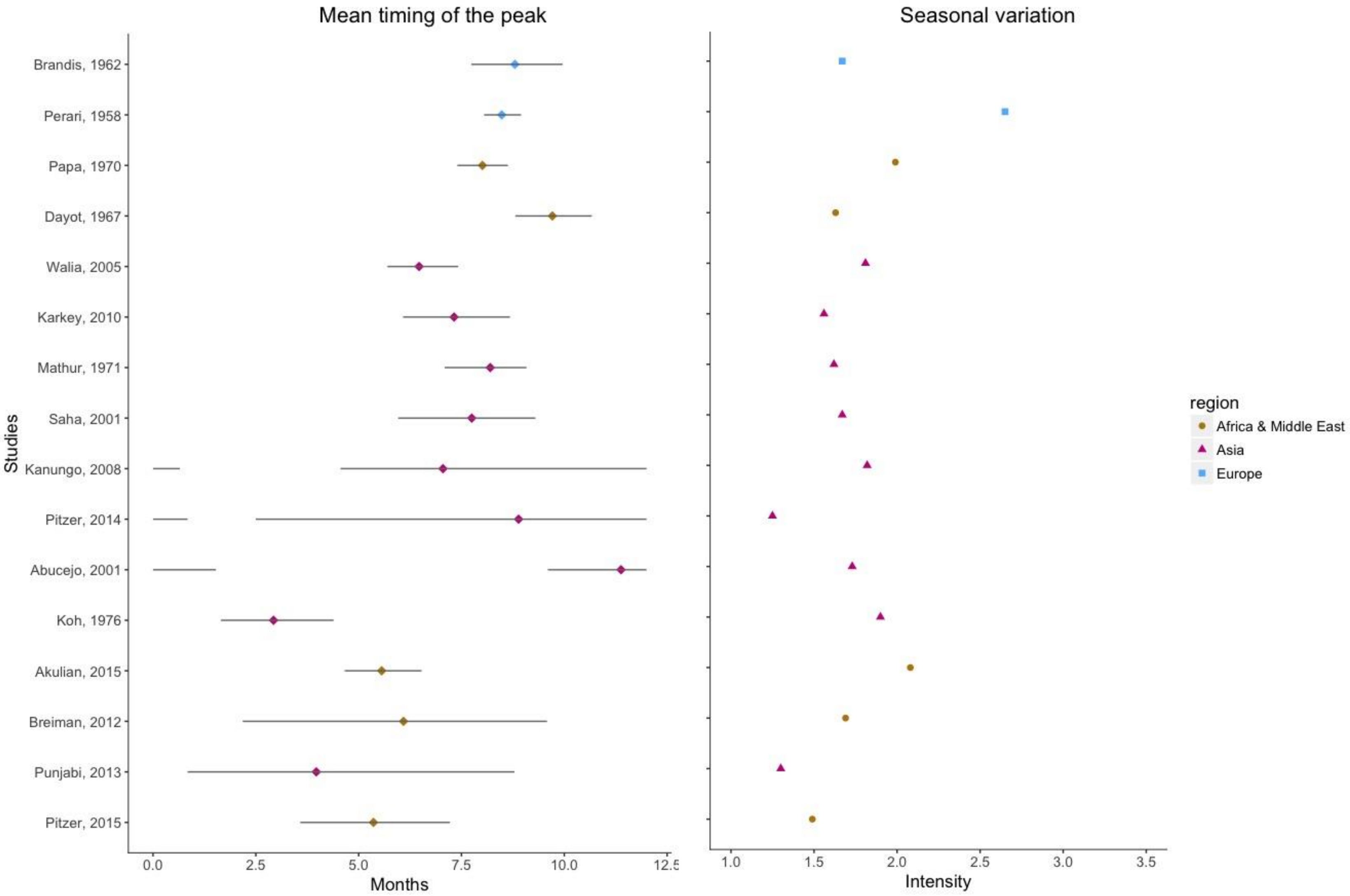

Supplementary Figure 12. Association of environmental drivers with seasonal intensity (peak/mean) and amplitude ((peak-trough)/peak) for data confirmed by blood or bone marrow culture only. Coefficients from the meta-regression of the seasonal intensity (peak/mean) and amplitude ((peak-trough)/peak) against the average location-specific rainfall and temperature, the variation in average rainfall and temperature (peak/mean), and the study location's elevation for all datasets with subnational data are plotted. The point estimates of the regression coefficients are represented by dots, while the 95% confidence interval is indicated by a horizontal line.

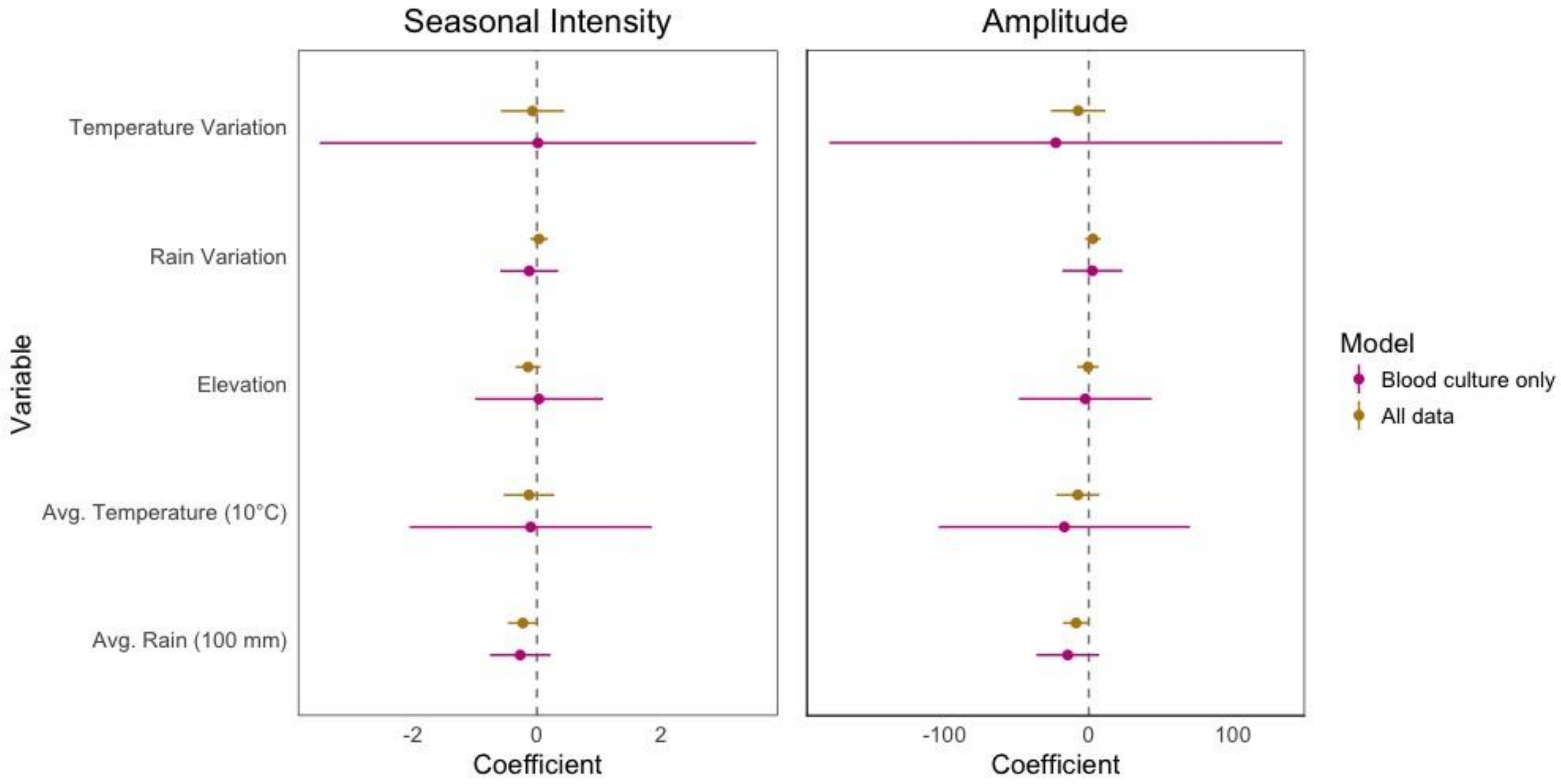

Supplementary Figure 13. Association of enteric or typhoid fever with environmental drivers for all studies and studies confirmed by blood or bone marrow culture only. The Pearson's correlation coefficient between enteric or typhoid fever and average monthly rainfall and average monthly temperature (current and lagged, up to 11 months) is summarized for all studies within a specified region. The values of the studies are represented in a boxplot, in which the whiskers represent the interquartile range (25th -75th percentile) times 1.5.

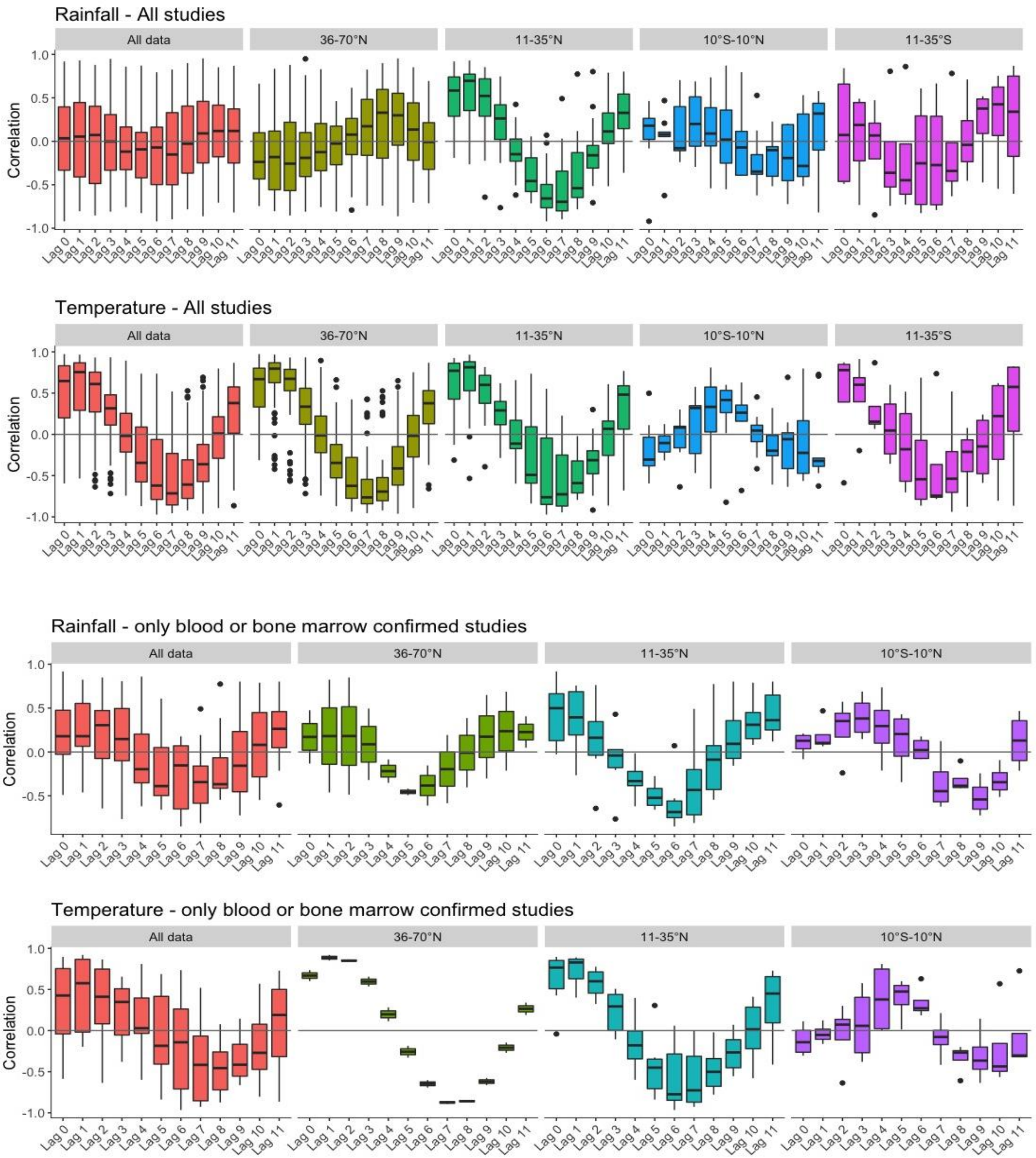

Table S1. Search strategy. We searched Embase, MEDLINE, Web of Science, PubMed and Global Health on the 16th of May 2016. We used both free text (FT) and controlled vocabulary (CV), except for Web of Science and PubMed where CV is not available. We used terms related to (para)typhoid and climatic variables (water/rain, temperature/heat, extreme weather events and climate/weather/season). We used Ovid to conduct the search of Embase, MEDLINE and Global Health and also restricted the search in these database to humans.

**Embase (Ovid)**	**MEDLINE (Ovid)**	**Web of Science**	**PubMed**	**Global Health (Ovid)**
**Database: Embase 1974 to 2016 May 13**	**Database: Medline 1946 to May Week 1 2016**	**Database: Web of Science Core collection**	**Database: Medline (index/non-indexed) and PubMed central**	**Database: Global Health 1910 to 2016 Week 18**
**(PARA)TYPHOID**<br>**FT:** (typhoid* or abdominal adj2 typhus or enteric adj2 fever* or typhus abdominalis).mp.<br>*or*<br>**FT**: (paratyphoid* or paratyphus).mp.<br>*or*<br>**FT**: (Salmonella enterica or Salmonella typhi or Salmonella typhosa or Salmonella paratyphi).mp.<br>*or*	**(PARA)TYPHOID**<br>**FT**: ((typhoid*) or (abdominal adj2 typhus) or (enteric adj2 fever*) or (typhus abdominalis)).mp.<br>or<br>**FT**: (paratyphoid* or paratyphus).mp.<br>or<br>**FT**: (Salmonella enterica or Salmonella typhi or Salmonella typhosa or Salmonella paratyphi).mp.<br>*or*	**(PARA)TYPHOID**<br>**FT:** (typhoid* or abdominal near/2 typhus or enteric near/2 fever* or typhus abdominalis)<br>*or*<br>**FT**: (paratyphoid* or paratyphus)<br>*or*<br>**FT**: (Salmonella enterica or Salmonella typhi or Salmonella typhosa or Salmonella paratyphi)<br>**AND**<br>**CLIMATIC VARIABLES**	**(PARA)TYPHOID**<br>**FT:** (typhoid* or abdominal adj2 typhus or enteric adj2 fever* or typhus abdominalis)<br>*or*<br>**FT**: (paratyphoid* or paratyphus)<br>*or*<br>**FT**: (Salmonella enterica or Salmonella typhi or Salmonella typhosa or Salmonella paratyphi)<br>**AND**<br>**CLIMATIC VARIABLES**	**(PARA)TYPHOID**<br>**FT**: ((typhoid*) or (abdominal adj2 typhus) or (enteric adj2 fever*) or (typhus abdominalis)).mp.<br>*or*<br>**FT**: (paratyphoid* or paratyphus).mp.<br>or<br>**FT**: (Salmonella enterica or Salmonella typhi or Salmonella typhosa or Salmonella paratyphi).mp.<br>*or*

**CV**: exp salmonella enterica/ or typhoid fever/ or paratyphoid fever/<br>**AND**<br>**CLIMATIC VARIABLES**<br>**WATER/RAIN**<br>**FT**: (rain* or water* or groundwater* or freshwater* or runoff or run-off or humid* or moist* or river* or ocean* or sea* or lake* or pond or ponds).mp.<br>*or*<br>**CV**: rain/ or humidity/ or water/ or groundwater/ or freshwater/ or runoff/ or water vapour/ or water flow/ or river/ or river water/ or “stream (river)”/ or ocean current/ or sea/ or lake/ or lake water/<br>**TEMPERATURE/ HEAT**<br>**FT**: (temperature or heat or warmth).mp.<br>*or*<br>**CV**: exp temperature/ or heat/	**CV**: exp salmonella enterica/ or Typhoid Fever/ or Paratyphoid Fever/<br>**AND**<br>**CLIMATIC VARIABLES**<br>**WATER/RAIN**<br>**FT**: (rain* or water* or groundwater* or freshwater* or runoff or run-off or humid* or moist* or river* or ocean* or sea* or lake* or pond or ponds).mp.<br>*or*<br>**CV**: "Oceans and Seas"/ or Rain/ or Humidity/ or Water/ or groundwater/ or exp freshwater/ or Water Movements/<br>**TEMPERATURE/ HEAT**<br>**FT**: (temperature* or heat or warmth).mp.<br>*or*<br>**CV**: exp Temperature/	**WATER/RAIN**<br>**FT**: (rain* or water* or groundwater* or freshwater* or runoff or run-off or humid* or moist* or river* or ocean* or sea* or lake* or pond or ponds)<br>*Or*<br>**TEMPERATURE/ HEAT**<br>**FT:** (temperature or heat or warmth)<br>Or<br>**EXTREME WEATHER EVENTS**<br>**FT:** (storm* or cyclon* or hurricane* or monsoon* or typhoon* or flood* or drought*)<br>*Or*<br>**CLIMATE/ WEATHER/ SEASON**	**WATER/RAIN**<br>(rain* or water* or groundwater* or freshwater* or runoff or run-off or humid* or moist* or river* or ocean* or sea* or lake* or pond or ponds)<br>*Or*<br>**TEMPERATURE/ HEAT**<br>**FT:** (temperature or heat or warmth)<br>*Or*<br>**EXTREME WEATHER EVENTS**<br>**FT:** (storm* or cyclon* or hurricane* or monsoon* or typhoon* or flood* or drought*)<br>*Or*<br>**CLIMATE/ WEATHER/ SEASON**	**CV**: exp "salmonella enterica subsp. enterica"/or typhoid/ or paratyphoid/<br>**AND**<br>**CLIMATIC VARIABLES**<br>**WATER/RAIN**<br>**FT**: (rain* or water* or groundwater* or freshwater* or runoff or run-off or humid* or moist* or river* or ocean* or sea* or lake* or pond or ponds).mp.<br>*or*<br>**CV**: rain/ or humidity/ or moisture/ or water/ or groundwater/ or runoff/ or coastal water/ or water vapour/ or water flow/ or rivers/ or river water/ or streams/ or oceans/ or seas/ or lakes/<br>**TEMPERATURE/ HEAT**<br>**FT**: (temperature or heat or warmth).mp.<br>*or*

**EXTREME WEATHER EVENTS**<br>**FT**: (storm* or cyclon* or hurricane* or monsoon* or typhoon* or flood* or drought*).mp.<br>*or*<br>**CV**: storm water/ or hurricane/ or flooding/ or drought/<br>**CLIMATE/ WEATHER/ SEASON**<br>**FT**: (weather or season* or (climate change) or cline* or El Nino* or La Nina*).mp.<br>*or*<br>**CV**: weather/ or season/ or climate/ or climate change/ or el nino/	**EXTREME WEATHER EVENTS**<br>FT: (storm* or cyclon* or hurricane* or monsoon* or typhoon* or flood* or drought*).mp.<br>*or*<br>**CV**: Cyclonic Storms/ or Floods/ or Droughts/<br>**CLIMATE/ WEATHER/ SEASON**<br>**FT**: (weather or season* or (climate change) or cline* or El Nino* or La Nina*).mp.<br>*or*<br>**CV**: Weather/ or Seasons/ or Climate Change/ or el nino/	**FT:** (weather or season* or (climate change) or cline* or El Nino* or La Nina*)	**FT**: (weather or season* or (climate change) or cline* or El Nino* or La Nina*)	**CV**: exp temperature/ or heat/<br>**EXTREME WEATHER EVENTS**<br>**FT**: (storm* or cyclon* or hurricane* or monsoon* or typhoon* or flood* or drought*).mp.<br>*or*<br>**CV**: storms/ or hurricanes/ or monsoon/ or floods/ or flooding/ or drought/<br>**CLIMATE/ WEATHER/ SEASON**<br>**FT**: (weather or season* or (climate change) or cline* or El Nino* or La Nina*).mp.<br>*or*<br>**CV**: weather/ or seasonality/ or "seasonal variation"/ or climate change/ or el nino-southern oscillation/

Supplementary Table 2. Characteristics of the included datasets.

Africa & Middle East						
**Author, year**	**City/Region, Country**	**Time period**	**Incidence (annual average, per 100,000 population)**	**Disease**	**Case ascertainment**	**Method of detection**
Abdurrahman, 1979 [1]	Kaduna, Nigeria	1973-1974	-	Enteric fever	Review of hospital records	Blood, stool or urine culture
Afifi, 2005 [2]	Egypt	Mar 1999 - Oct 2003	-	Typhoid fever	Passive surveillance at hospitals	Blood culture or Widal test
Ako, 2009 (1) [3]	Douala, Cameroon	1995-2006	-	Typhoid fever	Review of hospital records	Not specified
Ako, 2009 (2) [3]	Douala, Cameroon	1995-2006	-	Typhoid fever	Review of hospital records	Not specified
Ako, 2009 (3) [3]	Douala, Cameroon	1995-2006	-	Typhoid fever	Review of hospital records	Not specified
Akullian, 2015 [4]	Nairobi, Kenya	2010-2011	-	Typhoid fever	Passive surveillance at health clinic	Blood culture
Authur, 2015 [5]	Sunyani, Ghana	2008-2011	-	Typhoid fever	Review of hospital records	Laboratory-confirmed
Badaru, 2014 [6]	Ejule, Nigeria	2011-2013	-	Typhoid fever	National or regional disease surveillance or records	Not specified
Battikhi, 2003 [7]	Jordan	1995-1999	2.8	Enteric fever	National or regional disease surveillance or records	Stool culture
Breiman, 2012 [8]	Kibera, Kenya	Mar 2007 - Feb 2009	247.5	Typhoid fever	Active population-based surveillance at health clinic	Blood culture
Dayot, 1967 [9]	Morocco	1959-1962	-	Enteric fever	National or regional disease surveillance or records	Blood culture
Hamze, 2004 [10]	Tripoli, Libanon	1996-1999	-	Typhoid fever	Review of hospital records	Widal test
Hornus, 1934 (3) [11]	Morocco	1921-1930	13.6	Typhoid fever	National or regional disease surveillance or records	Not specified
Hornus, 1934 (4) [11]	Levant region (Libanon)	1921-1930	13.9	Typhoid fever	National or regional disease surveillance or records	Not specified
Hornus, 1934 (5) [11]	Tunesia & Algeria	1921-1930	3.08	Typhoid fever	National or regional disease surveillance or records	Not specified

Author, year	City/Region, Country	Time period	Incidence (annual average, per 100,000 population)	Disease	Case ascertainment	Method of detection
Kligler, 1933 (4) [12]	Israel and Palestine	1923-1931	-	Typhoid fever	Review of hospital records	Not specified
Naji-Rammal, 2010 [13]	Libanon	2000-2008	-	Enteric fever	National or regional disease surveillance or records	Blood, bone marrow or stool culture
Papa, 1970 [14]	El Oued, Algeria	Oct 1965 - Aug 1970	-	Enteric fever	Passive surveillance at health clinics	Blood culture
Pitzer, 2015 & Feasey, 2015 [15,16]	Blantyre, Malawi	Jan 2011 - Feb 2015	115.4	Typhoid fever	Passive surveillance at hospital	Blood culture
Valmary, 1979 [17]	Antananarivo, Madagascar	1976-1978	-	Typhoid fever	Review of hospital records	Blood or stool culture
Wicks, 1971 [18]	Harare, Zimbawe	1966-1969	-	Typhoid fever	Review of hospital records	Widal test, during operation or post-mortem
**Asia**						
Abucejo, 2001 [19]	Bohol, Philippines	Apr 1994 - Dec 1997	45	Typhoid fever	Review of hospital records	Blood culture
Dewan, 2013 [20]	Dhaka, Bangladesh	2005-2009	10	Typhoid fever	Review of hospital records	Blood culture or Widal test
Das, 2013 [21]	Dhaka, Bangladesh	1993-2012	-	Enteric fever	Review of hospital records	Stool culture
Hernando, 1937 [22]	Manilla, Phillippines	1915-1935	154.5	Typhoid fever	National or regional disease surveillance or records	Not specified
Kanungo, 2008 [23]	Kolkata, India	May 2003 - Aug 2006	-	Typhoid fever	Passive surveillance in health centres and hospitals	Blood culture
Karkey, 2010 [24]	Kathmandu, Nepal	1993-2011	59	Typhoid fever	Review of hospital records	Blood culture
Koh, 1976 [25]	Singapore	1970-1974	9.32	Enteric fever	National or regional disease surveillance or records	Blood culture
Liu, 2012 [26]	Linyi, China	1956-2011	2.5	Enteric fever	National or regional disease surveillance or records	Not specified
Mathur, 1971 [27]	Jaipur, India	1960-1969	46.8	Enteric fever	Review of hospital records	Blood culture

Author, year	City/Region, Country	Time period	Incidence (annual average, per 100,000 population)	Disease	Case ascertainment	Method of detection
Pan, 2012 [28]	Henan province, China	2004-2011	0.15	Typhoid fever	National or regional disease surveillance or records	Not specified
Patnaik, 1967 [29]	New Delhi, India	1955-1963	-	Typhoid fever	Review of hospital records	Not specified
Punjabi, 2013 [30]	Jakarta, Indonesia	Aug 2001 - Jul 2003	140	Typhoid fever	Passive surveillance at health centres	Blood culture
Farrar (unpublished data)	Vietnam	1977-1993	-	Typhoid fever	Review of hospital records	Clinical suspicion and blood culture
Qu, 2011 [31]	Guangzhou, Shenzhen, and Foushan cities in Guangdong province, China	2008-2010	-	Typhoid fever	National or regional disease surveillance or records	Not specified
Saha, 2001 [32]	Dhaka, Bangladesh	May 1997 - Nov 1999	-	Typhoid fever	Self-referred patients at a diagnositic facility and patients referred to the facility by hospitals, health centres, or communicity-based physicians	Blood culture
Siddiqui, 2006 [33]	Karachi, Pakistan	Jun 1999 - Dec 2001	-	Enteric fever	Patients referred to hospital after occurrence of febrile episode lasting >72h, which was ascertained by fortnightly visits to the households	Blood culture or Typhidot test
Tong, 2009 [34]	Lin'An, Zhejiang province, China	2002-2008	29.4	Enteric fever	National or regional disease surveillance or records	Not specified
Walia, 2005 [35]	New Dehli, India	2001-2003	980	Enteric fever	Review of hospital records	Blood culture
Wang, 2013 [36]	Hongta, China	2006-2009	26.83	Enteric fever	National or regional disease surveillance or records	Blood or stool culture
Wang, 2014 [37]	Guangxi, China	2010-2013	6.29	Typhoid fever	National or regional disease surveillance or records	Urine or stool culture or Widal test

Author, year	City/Region, Country	Time period	Incidence (annual average, per 100,000 population)	Disease	Case ascertainment	Method of detection
Wang, 2008 [38]	Yuxi, Yunnan province, China	1999-2006	130	Enteric fever	National or regional disease surveillance or records	Not specified
Xu, 2009 [39]	Nanning city, Guangxi province, China	2004-2007	0.82	Typhoid fever	National or regional disease surveillance or records	Not specified
Yang, 2004 [40]	22 counties in Guangxi Province, China	1994-2002	-	Typhoid fever	National or regional disease surveillance or records	Urine or stool culture or Widal test
Yao, 2014 [41]	Pingba county, Guizhou Province, China	2005-2008	51.26	Enteric fever	National or regional disease surveillance or records	Not specified
**Europe**						
Brandis, 1962 [42]	Hildesheim, Germany	1946-1960	14.1	Typhoid fever	National or regional disease surveillance or records	Blood culture
Camporese, 1960 (1) [43]	Grosseto province, Italy	1942 - 1949	105	Typhoid fever	National or regional disease surveillance or records	Not specified
Camporese, 1960 (2) [43]	Grosseto province, Italy	1950- 1957	20.6	Typhoid fever	National or regional disease surveillance or records	Not specified
Capasso, 1980 [44]	Bari, Italy	1955-1978	-	Enteric fever	National or regional disease surveillance or records	Not specified
Cominelli, 1971 [45]	Italy	1966-1967	-	Typhoid fever	National or regional disease surveillance or records	Not specified
Hornus, 1934 (1) [11]	France	1921-1930	12.4	Typhoid fever	National or regional disease surveillance or records	Not specified
Hornus, 1934 (2) [11]	Rhine region, France	1921-1929	3.4	Typhoid fever	National or regional disease surveillance or records	Not specified
Kligler, 1933 (1) [12]	England	1927-1929	-	Enteric fever	Review of hospital records	Not specified
Kligler, 1933 (2) [12]	Germany	1927-1929	-	Enteric fever	Review of hospital records	Not specified
Kligler, 1933 (3) [12]	Italy	1927-1929	-	Enteric fever	Review of hospital records	Not specified

Author, year	City/Region, Country	Time period	Incidence (annual average, per 100,000 population)	Disease	Case ascertainment	Method of detection
Moscato, 1976 (1) [46]	Italy	1955-1957	46.1	Enteric fever	National or regional disease surveillance or records	Not specified
Moscato, 1976 (2) [46]	Italy	1963-1965	25.1	Enteric fever	National or regional disease surveillance or records	Not specified
Moscato, 1976 (3) [46]	Italy	1969-1971	22.5	Enteric fever	National or regional disease surveillance or records	Not specified
Moscato, 1976 (4) [46]	Emilia Romagna, Italy	1955-1957	18.3	Enteric fever	National or regional disease surveillance or records	Not specified
Moscato, 1976 (5) [46]	Emilia Romagna, Italy	1963-1965	7.8	Enteric fever	National or regional disease surveillance or records	Not specified
Moscato, 1976 (6) [46]	Emilia Romagna, Italy	1969-1971	6.8	Enteric fever	National or regional disease surveillance or records	Not specified
Moscato, 1976 (7) [46]	Liguria, Italy	1955-1957	31.2	Enteric fever	National or regional disease surveillance or records	Not specified
Moscato, 1976 (8) [46]	Liguria, Italy	1963-1965	19.1	Enteric fever	National or regional disease surveillance or records	Not specified
Moscato, 1976 (9) [46]	Liguria, Italy	1969-1971	16.8	Enteric fever	National or regional disease surveillance or records	Not specified
Moscato, 1976 (10) [46]	Lazio, Italy	1955-1957	42.7	Enteric fever	National or regional disease surveillance or records	Not specified
Moscato, 1976 (11) [46]	Lazio, Italy	1963-1965	19.9	Enteric fever	National or regional disease surveillance or records	Not specified
Moscato, 1976 (12) [46]	Lazio, Italy	1969-1971	18.8	Enteric fever	National or regional disease surveillance or records	Not specified
Moscato, 1976 (13) [46]	Tuscany, Italy	1955-1957	19.4	Enteric fever	National or regional disease surveillance or records	Not specified

Author, year	City/Region, Country	Time period	Incidence (annual average, per 100,000 population)	Disease	Case ascertainment	Method of detection
Moscato, 1976 (14) [46]	Tuscany, Italy	1963-1965	12.2	Enteric fever	National or regional disease surveillance or records	Not specified
Moscato, 1976 (15) [46]	Tuscany, Italy	1969-1971	9.6	Enteric fever	National or regional disease surveillance or records	Not specified
Moscato, 1976 (16) [46]	Sardinia, Italy	1955-1957	94.1	Enteric fever	National or regional disease surveillance or records	Not specified
Moscato, 1976 (17) [46]	Sardinia, Italy	1963-1965	40	Enteric fever	National or regional disease surveillance or records	Not specified
Moscato, 1976 (18) [46]	Sardinia, Italy	1969-1971	38.4	Enteric fever	National or regional disease surveillance or records	Not specified
Moscato, 1976 (19) [46]	Sicily, Italy	1955-1957	65.2	Enteric fever	National or regional disease surveillance or records	Not specified
Moscato, 1976 (20) [46]	Sicily, Italy	1963-1965	31.9	Enteric fever	National or regional disease surveillance or records	Not specified
Moscato, 1976 (21) [46]	Sicily, Italy	1969-1971	28.6	Enteric fever	National or regional disease surveillance or records	Not specified
Moscato, 1976 (22) [46]	Campania, Italy	1955-1957	59.6	Enteric fever	National or regional disease surveillance or records	Not specified
Moscato, 1976 (23) [46]	Campania, Italy	1963-1965	33.9	Enteric fever	National or regional disease surveillance or records	Not specified
Moscato, 1976 (24) [46]	Campania, Italy	1969-1971	52.5	Enteric fever	National or regional disease surveillance or records	Not specified
Moscato, 1976 (25) [46]	Apulia, Italy	1955-1957	172	Enteric fever	National or regional disease surveillance or records	Not specified
Moscato, 1976 (26) [46]	Apulia, Italy	1963-1965	112.4	Enteric fever	National or regional disease surveillance or records	Not specified

Author, year	City/Region, Country	Time period	Incidence (annual average, per 100,000 population)	Disease	Case ascertainment	Method of detection
Moscato, 1976 (27) [46]	Apulia, Italy	1969-1971	77.8	Enteric fever	National or regional disease surveillance or records	Not specified
Moscato, 1976 (28) [46]	Basilicata, Italy	1955-1957	85.9	Enteric fever	National or regional disease surveillance or records	Not specified
Moscato, 1976 (29) [46]	Basilicata, Italy	1963-1965	81.3	Enteric fever	National or regional disease surveillance or records	Not specified
Moscato, 1976 (30) [46]	Basilicata, Italy	1969-1971	70.2	Enteric fever	National or regional disease surveillance or records	Not specified
Moscato, 1976 (31) [46]	Calabria, Italy	1955-1957	38.3	Enteric fever	National or regional disease surveillance or records	Not specified
Moscato, 1976 (32) [46]	Calabria, Italy	1963-1965	17.8	Enteric fever	National or regional disease surveillance or records	Not specified
Moscato, 1976 (33) [46]	Calabria, Italy	1969-1971	10.9	Enteric fever	National or regional disease surveillance or records	Not specified
Moscato, 1976 (34) [46]	Lombardy, Italy	1955-1957	20.6	Enteric fever	National or regional disease surveillance or records	Not specified
Moscato, 1976 (35) [46]	Lombardy, Italy	1963-1965	10.4	Enteric fever	National or regional disease surveillance or records	Not specified
Moscato, 1976 (36) [46]	Lombardy, Italy	1969-1971	10	Enteric fever	National or regional disease surveillance or records	Not specified
Moscato, 1976 (37) [46]	Piedmont, Italy	1955-1957	13.6	Enteric fever	National or regional disease surveillance or records	Not specified
Moscato, 1976 (38) [46]	Piedmont, Italy	1963-1965	7.5	Enteric fever	National or regional disease surveillance or records	Not specified
Moscato, 1976 (39) [46]	Piedmont, Italy	1969-1971	9.8	Enteric fever	National or regional disease surveillance or records	Not specified

Author, year	City/Region, Country	Time period	Incidence (annual average, per 100,000 population)	Disease	Case ascertainment	Method of detection
Moscato, 1976 (40) [46]	Veneto, Italy	1955-1957	26.5	Enteric fever	National or regional disease surveillance or records	Not specified
Moscato, 1976 (41) [46]	Veneto, Italy	1963-1965	13.6	Enteric fever	National or regional disease surveillance or records	Not specified
Moscato, 1976 (42) [46]	Veneto, Italy	1969-1971	11.9	Enteric fever	National or regional disease surveillance or records	Not specified
Muller-Voigt, 1956 (1) [47]	Duisburg, Germany	1945-1950	-	Typhoid fever	National or regional disease surveillance or records	Not specified
Muller-Voigt, 1956 (2) [47]	Düsseldorf, Germany	1945-1946	-	Typhoid fever	National or regional disease surveillance or records	Not specified
Muller-Voigt, 1956 (3) [47]	Oberhausen, Germany	1945-1946	-	Typhoid fever	National or regional disease surveillance or records	Not specified
Paquet, 1931 [48]	Oise region, France	1911-1930	-	Typhoid fever	National or regional disease surveillance or records	Not specified
Perari, 1958 [49]	Perugia, Italy	1939-1954	158.4	Enteric fever	National or regional disease surveillance or records	Blood culture
Stoll, 1949 [50]	Budapest, Hungary	1950-1956	10.5	Typhoid fever	National or regional disease surveillance or records	Not specified
Stoppani, 1958 [51]	Perugia, Italy	1939-1955	67	Typhoid fever	National or regional disease surveillance or records	Not specified
Torelli, 1956 [52]	Trani, Italy	1939-1953	170	Typhoid fever	National or regional disease surveillance or records	Not specified
Trompeo, 1950 [53]	Turin, Italy	1945-1949	244	Enteric fever	National or regional disease surveillance or records	Not specified
von der Forst, 1929 [54]	Münster, Germany	1925-1931	-	Typhoid fever	National or regional disease surveillance or records	Not specified

Author, year	City/Region, Country	Time period	Incidence (annual average, per 100,000 population)	Disease	Case ascertainment	Method of detection
Weinfurter, 1972 [55]	Niederosterreich, Lower Austria region, Austria	January 1922 - October 1926	-	Typhoid fever	National or regional disease surveillance or records	Stool culture and Widal test
**North- and South-America**						
**Author, year**	**City/Region, Country**	**Time period**	**Incidence (annual average, per 100,000 population)**	**Disease**	**Case ascertainment**	**Method of detection**
Falk, 1926 [56]	Chicago, USA	1912-1925	-	Typhoid fever	National or regional disease surveillance or records	Not specified
Harmon, 1930 (1) [57]	Northern States (Connecticut, Michigan, Minnnesota, New York and Wisconsin), United States of America	1915-1928	-	Typhoid fever	National or regional disease surveillance or records	Not specified
Harmon, 1930 (2) [57]	Southern States (Alabama, Louisiana, Mississippi), United States of America	1915-1928	-	Typhoid fever	National or regional disease surveillance or records	Not specified
Shuval, 1993 (1) [58]	Santiago, Chile	1973-1986	156.2	Typhoid fever	National or regional disease surveillance or records	Not specified
Shuval, 1993 (2) [58]	Chile	1973-1986	64.8	Typhoid fever	National or regional disease surveillance or records	Not specified

References

[1] Abdurrahman MB, Joss D V. Pattern of enteric fever in Kaduna, Nigeria. Niger Med J 1979;9:437–41.

[2] Afifi S, Earhart K, Azab MA, Youssef FG, El Sakka H, Wasfy M, et al. Hospital-based surveillance for acute febrile illness in Egypt: a focus on community-acquired bloodstream infections. Am J Trop Med Hyg 2005;73:392–9.

[3] Ako AA, Nkeng GE, Takem GEE. Water quality and occurrence of water-borne diseases in the Douala 4th District, Cameroon. Water Sci Technol 2009;59:2321–9. doi:10.2166/wst.2009.268.

[4] Akullian A, Ng'eno E, Matheson AI, Cosmas L, Macharia D, Fields B, et al. Environmental Transmission of Typhoid Fever in an Urban Slum. PLoS Negl Trop Dis 2015;9:e0004212. doi:10.1371/journal.pntd.0004212.

[5] Authur JL, Gyasi SF, Kabo-Bah AT, Awuah E. Rainfall variability and its impact on reported OPD cases of Salmonella typhi infections in Sunyani, Ghana. Res J Environ Sci 2015;9:39–47.

[6] Badaru YU, Olayemi IK, Spencer O, Yakubu M. Assessment of a vulnerable rural community to typhoid fever using geospatial-temporal analysis: case study of Ejule, Kogi state of Nigeria. J Environ Earth Sci 2014;4:100–8.

[7] Battikhi MNG. Occurrence of Salmonella typhi and Salmonella paratyphi in Jordan. New Microbiol 2003;26:363–73.

[8] Breiman RF, Cosmas L, Njuguna H, Audi A, Olack B, Ochieng JB, et al. Population-based incidence of typhoid fever in an urban informal settlement and a rural area in Kenya: Implications for typhoid vaccine use in Africa. PLoS One 2012;7. doi:10.1371/journal.pone.0029119.

[9] Dayot G, Nejmi S, Carraz M. Study of the epidemiology of Moroccan fevers. Rev Hyg Med Soc 1967;15:108–23.

[10] Hamze M, Vincent P. Typhoid fever in north Lebanon: a 8-year study (1992-1999) using the Widal test. East Mediterr Heal J 2004;10:180–6.

[11] Hornus G. Influence of Season on Epidemic Variations of Typhoid. Rev d'Hygiene Med Prev 1934;56:332–47.

[12] Kligler IJ, Olitzki L. The Epidemicity and Seasonal Prevalence of Infectious Diseases in Palestine. Folia Med Intern Orient 1933;1:169–94.

[13] Naji-Rammal S, Bedrossian N. Typhoid fever in Lebanon: epidemiological study. Sante n.d.;20:81–6. doi:10.1684/san.2010.0197.

[14] Papa F, Peyron R. A L'etude des Salmonella en Algerie 1970.

[15] Pitzer VE, Feasey NA, Msefula C, Mallewa J, Kennedy N, Dube Q, et al. Mathematical modeling to assess the drivers of the recent emergence of typhoid fever in Blantyre, Malawi. Clin Infect Dis 2015;61:S251–8. doi:10.1093/cid/civ710.

[16] Feasey NA, Gaskell K, Wong V, Msefula C, Selemani G, Kumwenda S, et al. Rapid Emergence of Multidrug Resistant, H58-Lineage Salmonella Typhi in Blantyre, Malawi. PLoS Negl Trop Dis 2015;9:1–13. doi:10.1371/journal.pntd.0003748.

[17] Valmary J, Capdevielle P, Thonnier C, Coign, Ard A. La Fievre Typhoide a Tananarive. Med Trop 1979;39:405–14.

[18] Wicks A, Holmes G, Davidson L. Endemic typhoid fever. Q J Med 1971;159:149–54.

[19] Abucejo PE, Capeding MR, Lupisan SP, Arcay J, Sombrero LT, Ruutu P, et al. Blood culture confirmed typhoid fever in a provincial hospital in the Philippines. Southeast Asian J Trop Med Public Health 2001;32:531–6.

[20] Dewan AM, Corner R, Hashizume M, Ongee ET. Typhoid Fever and Its Association with Environmental Factors in the Dhaka Metropolitan Area of Bangladesh: A Spatial and Time-Series Approach. PLoS Negl Trop Dis 2013;7:12–5. doi:10.1371/journal.pntd.0001998.

[21] Das SK, Chisti MJ, Malek MA, Shahnawaz A, Farzana FD, Farzana F, et al. Socio-demographic, host and clinical characteristics of people with typhoidal and non-typhiodal Salmonella gastroenteritis in urban Bangladesh. J Biomed Sci Eng 2013;6:834–42.

[22] Hernando E, Alomia A. Prevalence of Typhoid Fever in the City of Manila. Mon Bull Bur Heal 1937;17:79–109.

[23] Kanungo S, Dutta S, Sur D. Epidemiology of typhoid and paratyphoid fever in India. J Infect Dev Ctries 2008;2:454–60.
[24] Karkey A, Arjyal A, Anders KL, Boni MF, Dongol S, Koirala S, et al. The burden and characteristics of enteric fever at a healthcare facility in a densely populated area of kathmandu. PLoS One 2010;5. doi:10.1371/journal.pone.0013988.
[25] Koh TS, Goh KT. Enteric fever surveillance in Singapore. Singapore Med J 1976;17:32–7.
[26] Liu RT. Control counter-measures for eliminating epidemic periodicity of typhoid fever and paratyphoid fever in Linyi City. Occup Heal 2012;28:2273–5.
[27] Mathur GM, Sharma R. A study of typhoid fever in Jaipur, India. Trop Geogr Med 1971;23:329–34.
[28] Pan JJ, Xie ZQ, Kang K, Mu Y iao, Chen HM. Epidemiological characteristics of typhoid in Henan in 2004-2011. Chinese Prev Med 2012;13:767–9.
[29] Patnaik KC, Kapoor PN. A note on incidence of typhoid in Delhi. Indian J Med Res 1967;55:228–39.
[30] Punjabi NH, Agtini MD, Ochiai RL, Simanjuntak CH, Lesmana M, Subekti D, et al. Enteric fever burden in North Jakarta, Indonesia: a prospective, community-based study. J Infect Dev Ctries 2013;7:781–7.
[31] Qu Y Bin, Dai CF, Zhang JP. Correlation between water-borne diseases and qualified rate of total number of bacterial colonies in terminal tap water, Guangdong province. J Environ Heal 2011;28:1093–6.
[32] Saha SK, Baqui AH, Hanif M, Darmstadt GL, Ruhulamin M, Nagatake T, et al. Typhoid fever in Bangladesh: implications for vaccination policy. Pediatr Infect Dis J 2001;20:521–4.
[33] Siddiqui FJ, Rabbani F, Hasan R, Nizami SQ, Bhutta ZA. Typhoid fever in children: some epidemiological considerations from Karachi, Pakistan. Int J Infect Dis 2006;10:215–22. doi:10.1016/j.ijid.2005.03.010.
[34] Tong WS, Shan YM. Epidemiological feature of typhoid -paratyphoid fevers in Lin'an, Zhejiang province, 2002-2008. Dis Surveill 2009;24:349–51.
[35] Walia M, Gaind R, Mehta R, Paul P, Aggarwal P, Kalaivani M. Current perspectives of enteric fever: a hospital-based study from India. Ann Trop Paediatr 2005;25:161–74. doi:10.1179/146532805X58085.
[36] Wang JF, Wang Y, Zhang J, Christakos G, Sun JL, Liu X, et al. Spatiotemporal Transmission and Determinants of Typhoid and Paratyphoid Fever in Hongta District, Yunnan Province, China. PLoS Negl Trop Dis 2013;7:1–9. doi:10.1371/journal.pntd.0002112.
[37] Wang M, Kan B, Yang J, Lin M, Yan M, Zeng J, et al. Epidemiological characteristics of typhoid fever and antibiotic susceptibility testing of Salmonella Typhi isolates in Guangxi, 1994-2013. Chinese J Epidemiol 2014;35:930–4.
[38] Wang SY, Li K, Li XM. Epidemiological analysis of typhoid and paratyphoid in Hongta district of Yuxi city from 1999 to 2006. Dis Surveill 2008;23:415–6.
[39] Xu B, Huang X, Tang L. Analysis on epidemic characteristics of typhoid and paratyphoid fever in Nanning City in 2004-2007. China Trop Med 2009;9:516–7.
[40] Yang J, Dong BQ, Wang ML. Analysis of prevalent status of paratyphoid A and typhoid in Guangxi Autonomous Region in 1994-2002. China Trop Med 2004;4:177–8.
[41] Yao GH, Tang GP, Chen X, Tian KC, Zhang J, Sun JL, et al. Research on epidemiological features and risk factors of typhoid and paratyphoid fever in Pingba County, Guizhou Province. Mod Prev Med 2014;41:385–7.
[42] Brandis H, Walden M. The presence of typhoid and paratyphoid B in an administrative area from 1946 to 1960. Z Immun Exp Ther 1962;123:412–23.
[43] Camporese F, Martin Wedard A. Endemicity of Typhoid and Para-typhoid in Grosseto Province, 1942-57. Ig Sanita Pubbl 1960;16:159–203.
[44] Capasso V, Grosso E, Serio G. Mathematical models in epidemiological analysis. II. Typhoid fever: time series analysis. Ann Sclavo n.d.;22:189–206.
[45] Cominelli A. The course of typhoid and paratyphoid in recent years. Ann Sanita Pubblica 1971;32:357–61.
[46] Moscato V, Salvo S. The evolution of typhoid and paratyphoid in Italy. Some observations on the incidence by month of reported cases in the various regions. G Mal Infett Parassit 1976;28:635–44.
[47] Muller-Voigt F. A so far unknown typhoid epidemic of 1945-46 and its effects during the following

decade; epidemiological, hygienic and public health aspects. Offentl Gesundheitsdienst 1956;18:43–51.
[48] Paquet. Seasons and Rainfall and Infective Disease. Rev d'Hyg Med Prev 1931;53:401–22.
[49] Perari D. Survey of typhoid-para-typhoid infections within the municipal territory of Perugia during the period 1939-1954. Ann Sanita Pubblica n.d.;19:623–41.
[50] Stoll K. Typhoid fever epidemiology in Budapest. Nepegeszseguegy 1949;38:193–205.
[51] Stoppani A. The Incidence of Typhoid and Paratyphoid in Perugia Province in the Last 17 Years. G Mal Infett Parassit 1958;10:914–23.
[52] Torelli F. Observations on the Endemicity of Typhoid Fever in Trani in the 15 Years 1939-1953. G Mal Infett Parassit 1956;8:24–8.
[53] Trompeo G, Giacomasso P. Seasonal Recurrence of Enteric Fevers in Turin. G Accad Med Torino 1950;113:9–14.
[54] von der Forst P. The Incidence of Typhoid and Paratyphoid Fever, Dysentery and Food Poisoning in the Munster Area of Westphalia, 1925-31. Arch F Hyg U Bakt 1932;109:85–107.
[55] Weinfurter F. Typhoid Fever in Lower Austria in the Period after the War. Seuchenbekampfung 1927;4:131–9.
[56] Falk I. The Fatality of Typhoid Fever in Chicago. J Prev Med 1926;1:53–69.
[57] Harmon GE. Seasonal Distribution of Typhoid Fever-Southern and Northern States. Am J Public Health Nations Health 1930;20:395–402.
[58] Shuval HI. Investigation of typhoid fever and cholera transmission by raw wastewater irrigation in Santiago, Chile. Water Sci Technol 1993;27:167–74.

Supplementary Table 3. Comparison of typhoid and paratyphoid datasets.

Author, year	City/Region, Country	Time period	Typhoid incidence (annual average, per 100,000 population)	Paratyphoid incidence (annual average, per 100,000 population)	Region
Brandis, 1962 [1]	Hildesheim, Germany	1946-1960	14.1	7.0	Europe
Camporese, 1960 (1) [2]	Grosseto province, Italy	1942 - 1949	105	19.6	Europe
Camporese, 1960 (2) [2]	Grosseto province, Italy	1950- 1957	20.6	9.8	Europe
Cominelli, 1971 [3]	Italy	1966-1967	-	-	Europe
Kanungo, 2008 [4]	Kolkata, India	May 2003 - Aug 2006	-	-	Asia
Karkey, 2010 [5]	Kathmandu, Nepal	1993-2011	59	23	Asia
Kligler, 1933 [6]	Israel and Palestine	1923-1931	-	-	Africa & Middle East
Punjabi, 2013 [7]	Jakarta, Indonesia	Aug 2001 - Jul 2003	140	50	Asia
Qu, 2011 [8]	Guangzhou, Shenzhen, and Foushan cities in Guangdong province, China	2008-2010	-	-	Asia
Stoppani, 1958 [9]	Perugia, Italy	1939-1955	67	17	Europe
von der Forst, 1929 [10]	Münster, Germany	1925-1931	-	-	Europe
Xu, 2009 [11]	Nanning city, Guangxi province, China	2004-2007	0.82	0.44	Asia
Yang, 2004 [12]	22 counties in Guangxi Province, China	1994-2002	-	-	Asia

References

[1] Brandis H, Walden M. The presence of typhoid and paratyphoid B in an administrative area from 1946 to 1960. Z Immun Exp Ther 1962;123:412–23.

[2] Camporese F, Martin Wedard A. Endemicity of Typhoid and Para-typhoid in Grosseto Province, 1942-57. Ig Sanita Pubbl 1960;16:159–203.

[3] Cominelli A. The course of typhoid and paratyphoid in recent years. Ann Sanita Pubblica 1971;32:357–61.

[4] Kanungo S, Dutta S, Sur D. Epidemiology of typhoid and paratyphoid fever in India. J Infect Dev Ctries 2008;2:454–60.

[5] Karkey A, Arjyal A, Anders KL, Boni MF, Dongol S, Koirala S, et al. The burden and characteristics of enteric fever at a healthcare facility in a densely populated area of kathmandu. PLoS One 2010;5. doi:10.1371/journal.pone.0013988.

[6] Kligler IJ, Olitzki L. The Epidemicity and Seasonal Prevalence of Infectious Diseases in Palestine. Folia Med Intern Orient 1933;1:169–94.

[7] Punjabi NH, Agtini MD, Ochiai RL, Simanjuntak CH, Lesmana M, Subekti D, et al. Enteric fever burden in North Jakarta, Indonesia: a prospective, community-based study. J Infect Dev Ctries 2013;7:781–7.

[8] Qu Y Bin, Dai CF, Zhang JP. Correlation between water-borne diseases and qualified rate of total number of bacterial colonies in terminal tap water, Guangdong province. J Environ Heal 2011;28:1093–6.

[9] Stoppani A. The Incidence of Typhoid and Paratyphoid in Perugia Province in the Last 17 Years. G Mal Infett Parassit 1958;10:914–23.

[10] von der Forst P. The Incidence of Typhoid and Paratyphoid Fever, Dysentery and Food Poisoning in the Munster Area of Westphalia, 1925-31. Arch F Hyg U Bakt 1932;109:85–107.

[11] Xu B, Huang X, Tang L. Analysis on epidemic characteristics of typhoid and paratyphoid fever in Nanning City in 2004-2007. China Trop Med 2009;9:516–7.

[12] Yang J, Dong BQ, Wang ML. Analysis of prevalent status of paratyphoid A and typhoid in Guangxi Autonomous Region in 1994-2002. China Trop Med 2004;4:177–8.
